# Trends in Smart Restaurant Research: Bibliometric Review and Research Agenda

**DOI:** 10.12688/f1000research.158066.1

**Published:** 2024-12-09

**Authors:** Alejandro Valencia-Arias, Sebastián Cardona-Acevedo, Ezequiel Martínez Rojas, Juana Ramírez Dávila, Paula Rodriguez-Correa, Lucia Palacios-Moya, Renata Teodori de la Puente, Erica Agudelo-Ceballos, Martha Benjumea-Arias

**Affiliations:** 1 School of Industrial Engineering, Universidad Senor de Sipan, Chiclayo, Lambayeque, 14001, Peru; 2 Centro de Investigaciones Escolme-CIES, Institución Universitaria Escolme, Medellín, 050005, Colombia; 3 Vicerrectoría de Investigación e Innovación, Universidad Arturo Prat, Iquique, Tarapacá Region, Chile; 4 Departamento Estudios Generales, Universidad Senor de Sipan, Chiclayo, Lambayeque, Peru; 5 Escuela de posgrado, Universidad Senor de Sipan, Chiclayo, Lambayeque, Peru; 6 Instituto de Investigación y Estudios de la Mujer, Universidad Ricardo Palma, Santiago de Surco, Peru; 7 Departamento de Ciencias Administrativas, Instituto Tecnológico Metropolitano, Medellín, Colombia

**Keywords:** Smart restaurants; PRISMA; machine learning; deep learning; IoT.

## Abstract

**Background:**

The automation of processes and services has transformed various industries, including the restaurant sector. Technologies such as the Internet of Things (IoT), machine learning, Radio Frequency Identification (RFID), and big data have been increasingly adopted to enhance service delivery, improve user experiences, and enable data traceability. By collecting user feedback and analyzing sentiments, these technologies facilitate decision-making and offer predictive insights into future food preferences. This study aims to explore current research trends in intelligent restaurants, focusing on technological applications that improve service and decision-making.

**Methods:**

A bibliometric analysis was conducted in accordance with the PRISMA-2020 guidelines. A total of 94 academic documents were reviewed from the Scopus and Web of Science databases, focusing on publications related to intelligent restaurant systems, particularly involving IoT and automation technologies.

**Results:**

The analysis revealed that the United States, India, and China have contributed the most to the field, with a particular emphasis on China’s implementation of IoT architecture and robotics in restaurant settings. Chinese restaurant innovations, particularly in robotics, are among the most frequently cited in the literature. The study identifies these countries as leading the research in the intelligent restaurant domain.

**Conclusions:**

Technologies such as IoT, machine learning, RFID, and big data are driving advancements in restaurant automation, enhancing service efficiency and user experience. The United States, India, and China are leading research in this area, with China standing out for its application of robotics and IoT in restaurants. This research provides a foundation for future studies aimed at improving predictive models for food selection and service optimization.

## Introduction

The intensive use of various advanced technological tools in different economic sectors is becoming increasingly widespread. The development of information and communication technologies (ICT) has brought about significant changes in the way people interact and adapt to different social environments ( Bai & Wu, 2022). Thus, the maturity of technology has influenced the productivity and daily life of people who use these tools to have a better quality of life, greater efficiency in their daily activities, and to enjoy the convenience they have brought ( Bai & Wu, 2022).

Tools such as Artificial Intelligence (AI), the Internet of Things (IoT), building information, big data, cloud computing, machine learning ( Wu & Huang, 2020), and automation tools have allowed companies dedicated to different activities to achieve better results in terms of productivity, time, competitiveness, and economics ( Jubaid et al., 2023). The adoption of these technologies makes it possible to create new types of business architectures and build sustainable and intelligent industries ( Elkhwesky & Elkhwesky, 2023). One sector in which the adoption of technological tools has expanded is the tourism and services sector, especially in restaurants ( Singh et al., 2023).

In this sense, as users become more comfortable with technologies that allow, for example, self-service, automatic ordering and payment systems, and robotic services, this type of technology will continue to become more relevant in restaurants ( Wong et al., 2022). Some of these technologies became more relevant, especially in the context of Covid-19, where one of the most affected industries was restaurants, which, in order to adapt to the new reality, were forced to implement this type of technology to provide their services again ( Hasan et al., 2022).

However, some restaurants have continued to implement technologies to improve the user experience and update the way they provide the service. Some of the technologies that have been adapted include a personal digital assistant (PDA), Wi-Fi access, and high-end monitors for menu selection, including the use of holograms ( Kavitha et al., 2022). To adapt to the context of smart cities, restaurants are transforming into smart restaurants. For example, the use of augmented reality in menus can enhance the user’s dining experience and facilitate operations related to ordering and billing by automating this entire process ( Prathibanandhi et al., 2022).

The integration of elements such as cloud computing allows different services to be hosted in the cloud and has information available to customers and employees; the use of sensors and IoT devices allows restaurants to have better management of the restaurant, providing in-formation on the occupancy of the premises, interactive menus, availability of food, process and verification of food quality, and organization ( Martino et al., 2022). One of the advances that has had the greatest impact on restaurants is the use of robots for user service, either as waiters or just to take the order ( Mishra et al., 2022). However, the use of this type of technology requires the integration of different tools, such as Internet access services, control, monitoring, and identification services, to integrate people, things, and objects ( Wu & Huang, 2020).

In addition, it is possible to create interactive menus that can identify user preferences based on data collection, and through the use of chatbots, it is possible to collect purchase data and provide quick responses to consumers by making personalized recommendations ( Kaur et al., 2023).

However, in restaurants, robots need to understand the environment and make decisions accordingly, such as changing their route if they encounter obstacles along the way. To have the ability to make decisions and behave like humans, robots need information from multiple sources from which they can understand the change in the environment, the position of other robots, and the path to the destination. This can be achieved by integrating robots with IoT technology, which improves the capabilities of a robotic waiter ( Mishra et al., 2022).

The above not only allows for a personalized and more interactive experience for the user but also increases the possibility of meeting their expectations. This is because today’s users are much more demanding, have more options to choose from, and have more bargaining power in addition to being content generators who share their experiences with other users ( Almeida et al., 2023). On the other hand, it is also possible to support marketing strategies through the use of digital tools, allowing the restaurant’s competitive advantage to be improved using data from reviews on social networks, patterns of feelings, and behavior through the use of big data ( Hajek & Sahut, 2022).

Technology adoption in the restaurant sector remains low due to a variety of interrelated factors. first, resistance to change by restaurant owners and managers can be a significant barrier, many of which may be rooted in traditional practices. and may be reluctant to invest in technology due to concerns about cost, implementation complexity, and fear of operational disruption. and a lack of knowledge and understanding of how to effectively integrate technology into restaurant operations can lead to uncertainty and avoidance. adoption. It is suggested that the internal environmental locus of control, that is, the perception of control over the internal environment of the restaurant, can influence behavioral intentions toward technology adoption, highlighting the importance of addressing the attitudes and perceptions of customers. owners and managers ( Joo, Kim, and Hwang, 2023).

In addition, lack of access to adequate resources and training can be a limiting factor in the adoption of technology in the restaurant sector; owners and managers may lack the technical knowledge necessary to evaluate, select, and implement technological solutions more ap-propriate for their operations. in addition, the lack of adequate technological infrastructure, such as reliable Internet connectivity, can hinder the effective implementation of advanced technologies. The importance of factors such as consumer attitudes and loyalty towards smart vending technology in restaurants is highlighted, indicating that customer perceptions and experiences can also influence technology adoption by restaurant operators ( Lacap et al., 2023); these factors may contribute to low technology adoption in the restaurant sector by creating perceived and real barriers to the successful implementation of technological solutions.

Although the adoption of these technologies in various sectors has been widely addressed, the hospitality and tourism sector, which includes restaurants, still requires more research and the adoption of these technologies in this sector remains low. ( Elkhwesky and Elkhwesky, 2023; Klouvidaki et al., 2023). Therefore, research is needed to identify the factors and advances in the incorporation of technological elements that facilitate the development of smart restau-rants. In this sense, the objective of the research is to analyze research trends in the field of smart restaurants in order to identify areas that require greater attention and progress, this will be done through a comprehensive bibliometric review of the existing literature on the topic. The ultimate goal is to provide a global perspective of the prevailing approaches in the development of smart restaurants and to guide a research agenda for future studies in this area. By better understanding customer needs and preferences, as well as the benefits and challenges associated with the implementation of emerging technologies, this research seeks to provide clear guidance for decision-making and strategic planning in the sector.

In addition, this research also seeks to contribute at a theoretical level to the field of innovation and technology management in the hospitality industry by identifying relevant models and conceptual frameworks to understand and address the specific challenges faced by restaurants in the digital era. The results of this research are expected to not only benefit restaurant owners by providing them with valuable insights to improve their operations and services, but also contribute to the advancement of the food and beverage industry as a whole by fostering innovation and adoption of technologies that enhance competitiveness and long-term sustainability. Research can provide practical and strategic insights that help restaurants adapt to an ever-changing environment, improve their competitiveness, and meet rising customer expectations in the digital age. For this purpose, the following research questions were asked.

RQ1: What are the years of greatest interest in research on smart restaurants?

RQ2: What kind of growth exists in the number of scientific articles about research on smart restaurants?

RQ3: What are the main research references on smart restaurants?

RQ4: What is the thematic evolution of scientific production in smart restaurants?

RQ5: What are the main thematic clusters on smart restaurants?

RQ6: What are the growing and emerging keywords in Smart Restaurants research?

RQ7: Which topics are positioned as protagonists in the design of a research agenda on smart restaurants?

In this sense, the article is composed of a methodological section, which details the way the article is developed, as well as the sources of information, the results or findings obtained, their discussion, as well as the identification of research gaps, the approach to the research agenda, and, finally, the main conclusions.

## Methods

As part of this exploratory study on smart restaurants, a bibliometric analysis was conducted using the guidelines described in the PRISMA-2020 statement. ( Page et al., 2021; Valencia-Arias et al., 2024; García-Pineda et al., 2024). The collection and review of relevant secondary research materials forms the basis of this methodology.

### Eligibility criteria

The establishment of inclusion criteria that guarantee the selection of relevant texts for analysis is crucial in the context of this bibliometric study, which has Smart Restaurants as its main focus. In this sense, it was determined that one of the exclusion criteria is related to texts that do not directly contribute to the field of study, thus ensuring the quality and relevance of the documents considered in the bibliometric analysis.

The document selection and exclusion processes consisted of three distinct phases. In the first phase, all records with indexing errors were discarded to ensure the integrity of the biblio-metric database. Only systematic literature reviews are subject to the second phase of exclusion, which involves eliminating all documents for which the full text is not available, as these reviews require an exhaustive examination of the content. It should be noted that bibliometrics is currently based only on the evaluation of available metadata. To ensure that only relevant documents were analyzed in this bibliometric research, texts that did not meet the previously established relevance criteria were eliminated in the third phase of exclusion.

### Source of information

For this bibliometric study on smart restaurants, we used the Scopus and Web of Science databases, which are currently considered the main sources of academic and scientific in-formation. This decision was based on the comprehensive coverage of both databases, which covered a wide range of disciplines and provided access to numerous academic journals and conferences. This choice is further supported by a study comparing Scopus and Web of Science in a typical university environment, highlighting the value and effectiveness of both in searching and retrieving scientific literature. The use of these well-known databases guaran-tees that the information is up-to-date and relevant, thereby improving the precision and reliability of the data used in this bibliometric analysis ( Vieira & Gomes, 2009).

### Search strategy

To perform an efficient search, two specialized search equations were developed for the two selected databases, Scopus and Web of Science. These equations were carefully designed to meet the previously established inclusion criteria while also being tailored to the unique search requirements of each database. This strategy allowed them to retrieve relevant scientific literature and ensure the accuracy and integrity of the data collection for ongoing bibliometric research.

For the Scopus database: (TITLE (“Smart Restaurant*” OR “smart restaurant*" OR (iot AND restaurant*) OR (“internet of things” AND restaurant*) OR (“artificial intelligence” AND restaurant*) OR (“virtual reality” AND restaurant*) OR (“augmented reality” AND restaurant*)) OR KEY (“Smart restaurant*” OR “smart restaurant*”) OR (iot AND restaurant*) OR (“Internet of Things” AND restaurant*) OR (“Artificial Intelligence” AND restaurant*) OR (“Virtual Reality” AND restaurant*) OR (“Augmented Reality” AND restaurant*))).

For the Web of Science database: (TI=(“intelligent restaurant*” OR “smart restaurant*" OR (iot AND restaurant*) OR (“Internet of things” AND restaurant*) OR (“Artificial intelligence” AND restaurant*) OR (“Virtual reality” AND restaurant*) OR (“Augmented reality” AND restaurant*)) OR AK=(“Smart restaurant*” OR “intelligent restaurant*" OR (“IoT AND restaurant*”) OR (“Internet of Things AND restaurant*”) OR (“Artificial Intelligence AND restaurant*”) OR (“Virtual Reality AND restaurant*”) OR (“Augmented Reality AND restaurant*”))).

### Data management

For this bibliometric study on intelligent restaurants, the information obtained from each of the selected databases was extracted, stored, and processed using Microsoft Excel. This platform simplifies the data organization. The free VOSviewer ^®^ program was used in a similar manner ( Eck & Waltman, 2010). It allows the analysis and visualization of biblio-metric data and the creation of graphs representing different bibliometric indicators. In this study, the combination of Microsoft Excel ^®^ and VOSviewer ^®^ was found to be a crucial method for managing data and presenting results.

### Selection process

Based on the PRISMA 2020 guidelines ( Page et al., 2021). To determine the risk of omitting relevant studies or incorrectly classifying data, it is essential to answer the question of whether an internal automated classifier was used during the study selection and internal or external validation processes. These recommendations were followed in the present study, which used Microsoft Excel ^®^ automation tools as the internal mechanism for the selection process. All study investigators contributed to the development and improvement of this tool, which they then independently used to apply the inclusion and exclusion criteria. Through the convergence of the results of the application of the tool, this collaborative approach was adopted with the aim of minimizing the risk of omitting relevant studies or incorrect classifications, ensuring integrity and accuracy in the selection of studies for current restaurant bibliometrics. intelligent.

### Data collection process

Following the established guidelines, they described the procedures for obtaining or verifying data directly with the original investigators of the studies, any procedures for doing so, and, if applicable, details about the automation tools used in data collection ( Page et al., 2021). This includes the number of reviewers involved in the process, their degree of independence, and the procedures for doing so. These guidelines were strictly followed in the context of smart restaurant studies. To ensure efficiency and consistency in the extraction of information, Microsoft Excel ^®^ was used as an automated tool for data collection of the reports obtained from the two selected databases. In addition, all authors took on the role of reviewers and completed this task independently. Subsequently, a collective data validation process was implemented in which the authors worked together to validate and authenticate the results, ensuring a high level of consistency and reliability in the collection of bibliometric data.

### Data elements

An exhaustive search was conducted to find all relevant results and articles related to the research objective in the context of the current bibliometric research on smart restaurants. This was done using specific search equations created for each database, covering all articles that mentioned the topic of interest and ensuring that all relevant research was included. It is important to note that if any of the identified articles contain ambiguous or missing information, "non-relevant texts" are excluded from consideration. This was done to ensure that only findings that add to the understanding of the smart restaurant knowledge base and meet predefined relevance criteria are included to maintain consistency with the purpose and scope of the research.

### Study risk of bias assessment

All the authors worked diligently and collaboratively to collect data and assess the risk of bias in the included studies as part of the current bibliometric review of smart restaurants. Microsoft Excel ^®^, an automated tool, was used to ensure consistency and uniformity in the process. This method ensured the quality and integrity of the results obtained and contributed to the reliability of the ongoing bibliometric research by allowing a rigorous and standardized assessment of possible biases in the included studies.

### Effect measures

It is crucial to emphasize that effect measures that are more typical of primary research, such as hazard ratios and mean differences, are not appropriate in this example of source-based bibliometric research on smart restaurants. Instead, the results were analyzed and synthesized using bibliometric measures, including the number of publications, volume of citations, and timing of keyword use. The data were managed and analyzed using Microsoft Excel ^®^. Although not translated into measurements, VOSviewer ^®^ was also used to explore thematic associations by identifying nodes and networks, providing users with a deep understanding of the dynamics and evolution of research in the field of smart restaurants.

### Synthesis methods

A procedure was used in this bibliometric research on smart restaurants to select studies that could be included in the synthesis. This involved collecting information on the attributes of a document, such as the number of publications, citations, and keywords, and comparing them with the predetermined inclusion criteria. In addition, data preparation techniques were used, including imputing missing summary statistics and performing data conversion where necessary. Bibliometric indicators of quantity, quality, and structure were used in the presentation and synthesis of the results according to predetermined guidelines. These indicators were automatically applied to all documents that survived the three exclusion stages ( Durieux & Gevenois, 2010). Using this methodology, studies included in the bibliometric review were selected and analyzed for consistency and objectivity.

### Assessment of reporting bias

In the context of this bibliometric research on smart restaurants, it is important to be aware of possible information biases resulting from the use of synonyms found in thesauri, such as IEEE. These biases may be related to the lack of results in the synthesis. This vulnerability is reflected in the inclusion criteria, search approach, and data collection, where the choice of certain terms may affect the exclusion of relevant documents that use different vocabularies. Therefore, while bibliometrics must be accurate, the exclusion of irrelevant texts may result in the loss of important data for building comprehensive knowledge on the topic. This should be considered when interpreting the results and evaluating possible biases in bibliometric analyses.

### Assessment of certainty

**Figure 1.**
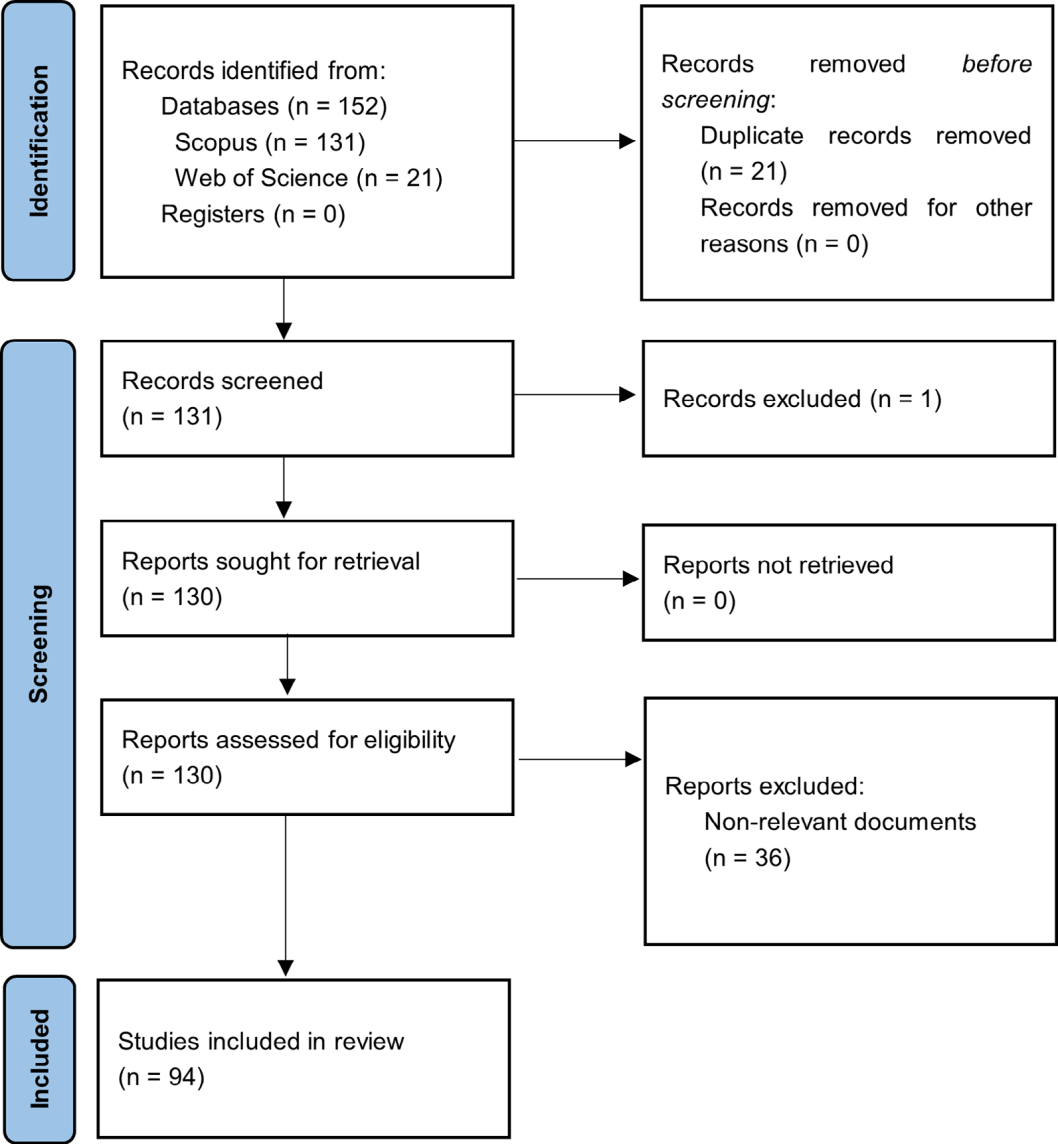
PRISMA flow chart. Own elaboration based on Scopus and Web of Science.

This bibliometric study on Smart Restaurants includes a comprehensive assessment of the certainty of the body of evidence. Here, certainty is assessed globally as opposed to primary studies, where certainty is assessed individually. This was achieved by applying independent inclusion and exclusion criteria, defining and analyzing bibliometric indicators, and identifying and disclosing potential biases resulting from the design of the methodology. In addition, the limitations of the study are discussed in the Discussion section, providing a com-prehensive assessment of the accuracy and reliability of the bibliometric results. This im-proves our understanding of the validity and substance of the conclusions. Figure 1 summarizes the methodological design.

In the first phase of this bibliometric study on Smart Restaurants, articles were identified based on the search strategy used for each selected information source, and duplicate records were eliminated. The three exclusion phases listed in the methodology section were then carried out by applying relevance and quality standards. After a long process, 94 articles that met the established criteria were identified and selected to form the main corpus of this bibliometric study.

## Results

**Figure 2.**
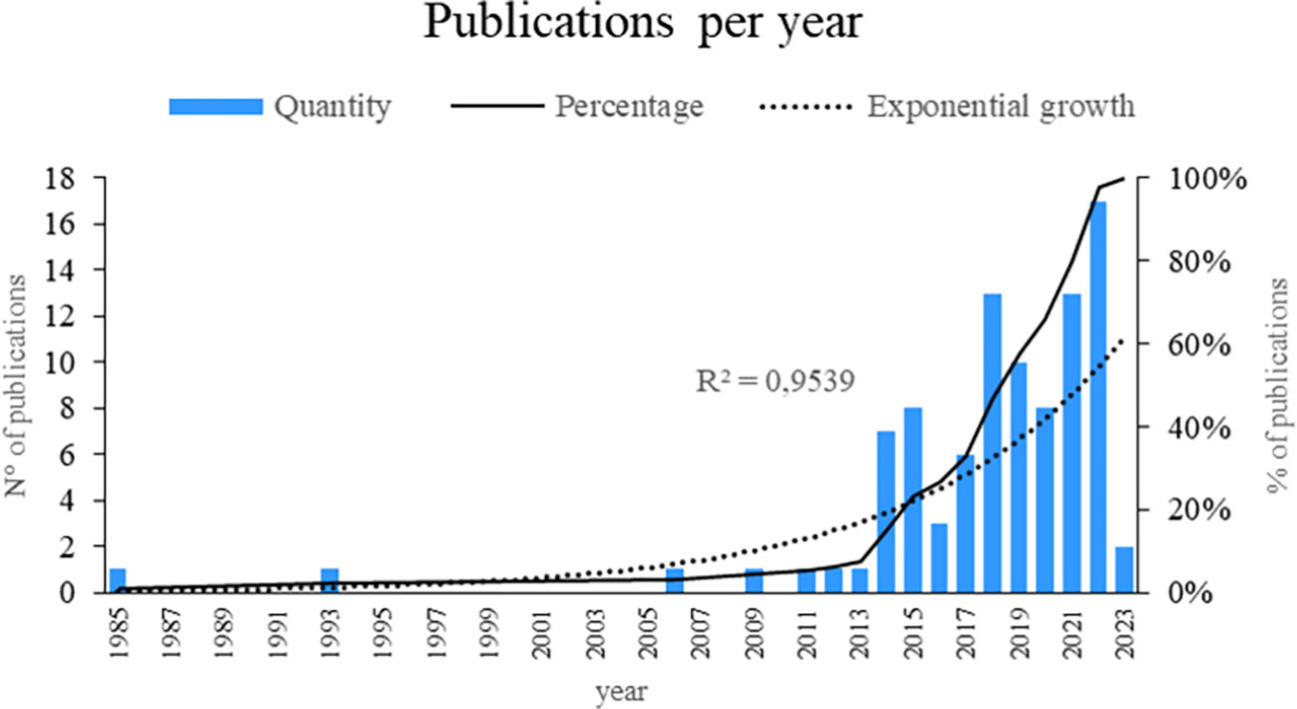
Publications by year. Own elaboration based on Scopus and Web of Science.

As shown in Figure 2, this bibliometric study provides a fascinating window for the evolution of smart restaurant research. The data showed a remarkable increase of 95.39 percent, suggesting an exponential growth pattern in the publication of articles related to this topic. Notably, the years with the highest number of publications are 2022, 2021, and 2018, suggesting that interest and research activities around smart restaurants increased significantly during these particular years. This may indicate the current relevance and timeliness of the topic in the scientific community. This temporal analysis sheds light on the changing nature of smart-restaurant research.

**Figure 3.**
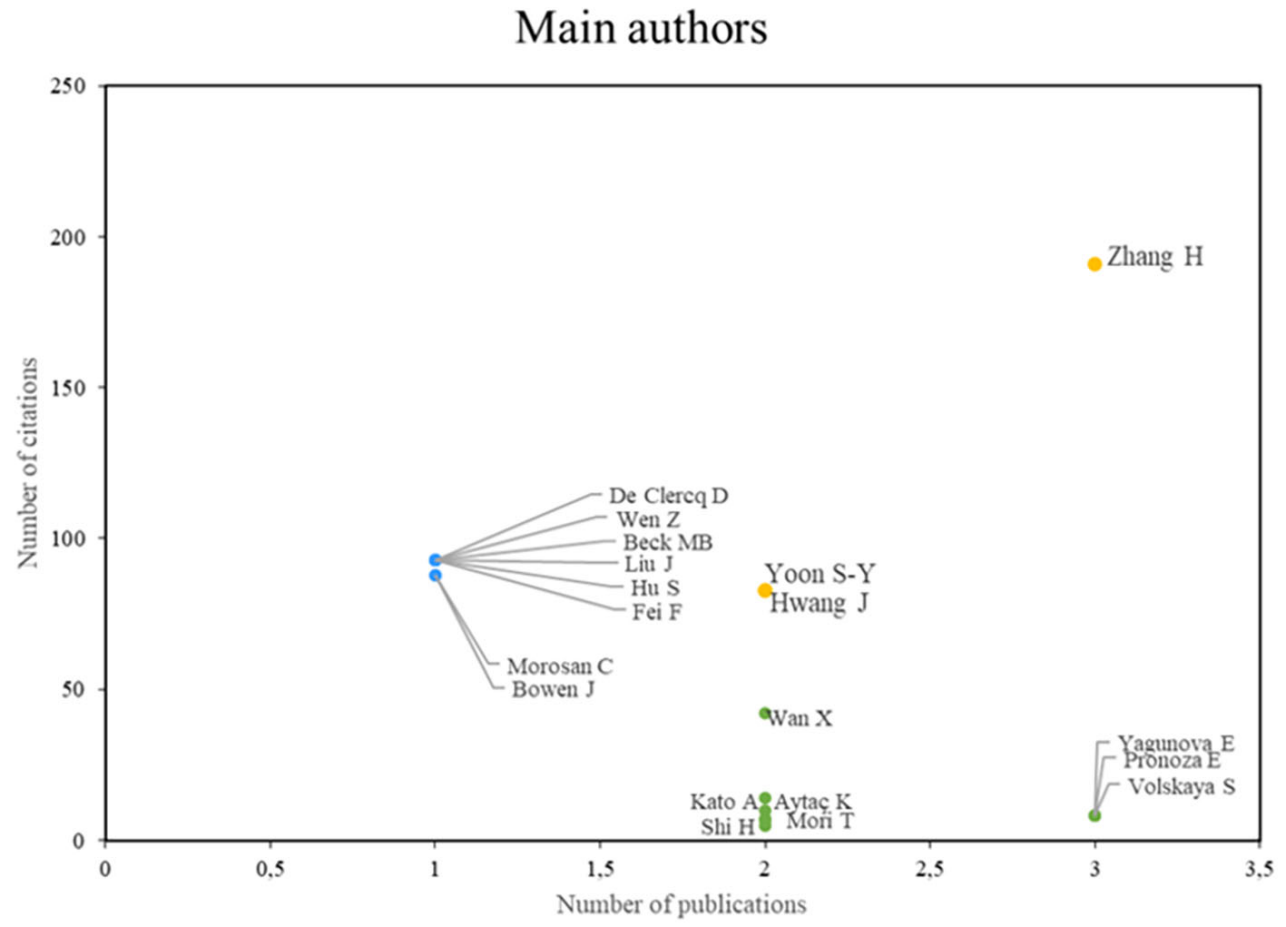
Main authors. Own elaboration based on Scopus and Web of Science.

According to the analysis of the main authors of this bibliometric study on smart restaurants, three groups can be identified. First, we can identify authors, such as Zhang, Yoon, and Hwang, who stand out in terms of their scientific productivity and the importance of their research, establishing them as leading figures in the field, as shown in Figure 3. Conversely, some authors (e.g., De Clercq, Wen, and Beck) value the impact and caliber of their work, even if their productivity rate is low. The third group of authors, Wan, Kato, and Aytac, for example, is characterized by high scholarly productivity, although not always in terms of citations, which enables them to actively advance our understanding of smart restaurants. This authorship analysis provides a deeper understanding of the current state of research in this field.

**Figure 4.**
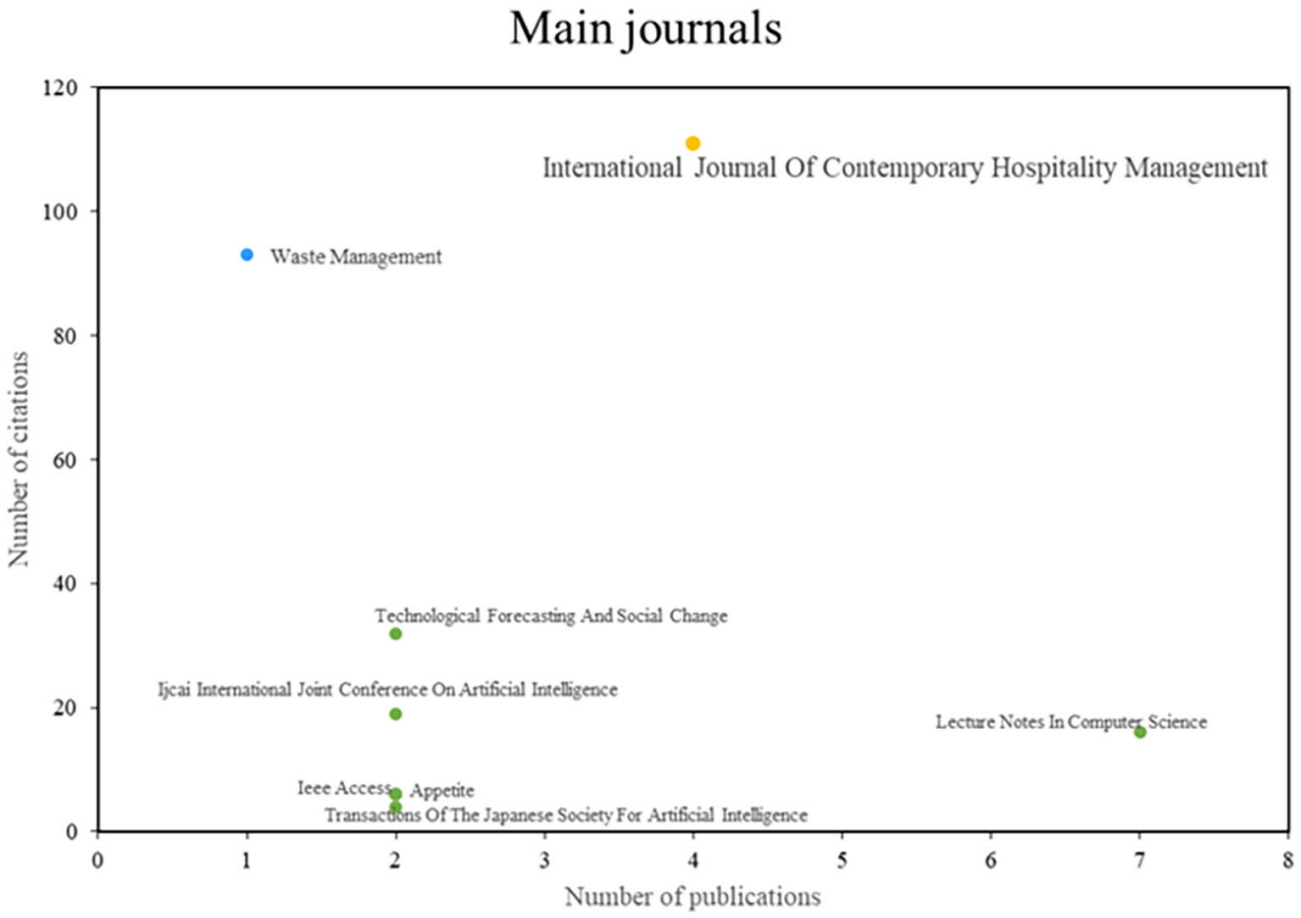
Major journals. Own elaboration based on Scopus and Web of Science.

After analyzing the main journals of this bibliometric on smart restaurants, they were able to observe three different groups (See Figure 4). First, publications such as the International Journal of Contemporary Hospitality Management stand out for their impact and productivity in the scientific community and have established themselves as leaders in the industry. On the other hand, there are publications on Waste Management, which are recognized for their impact and publication quality despite having a lower productivity index. Finally, the third group of journals is characterized by high scientific productivity, but the amount of research published rather than the number of citations is a better indicator of a journal’s influence. Examples of these journals are Technological Forecasting and Social Change and the Ijeai International Joint Conference on Artificial Intelligence. This journal analysis provides a deep understanding of the editorial dynamics in this research area.

**Figure 5.**
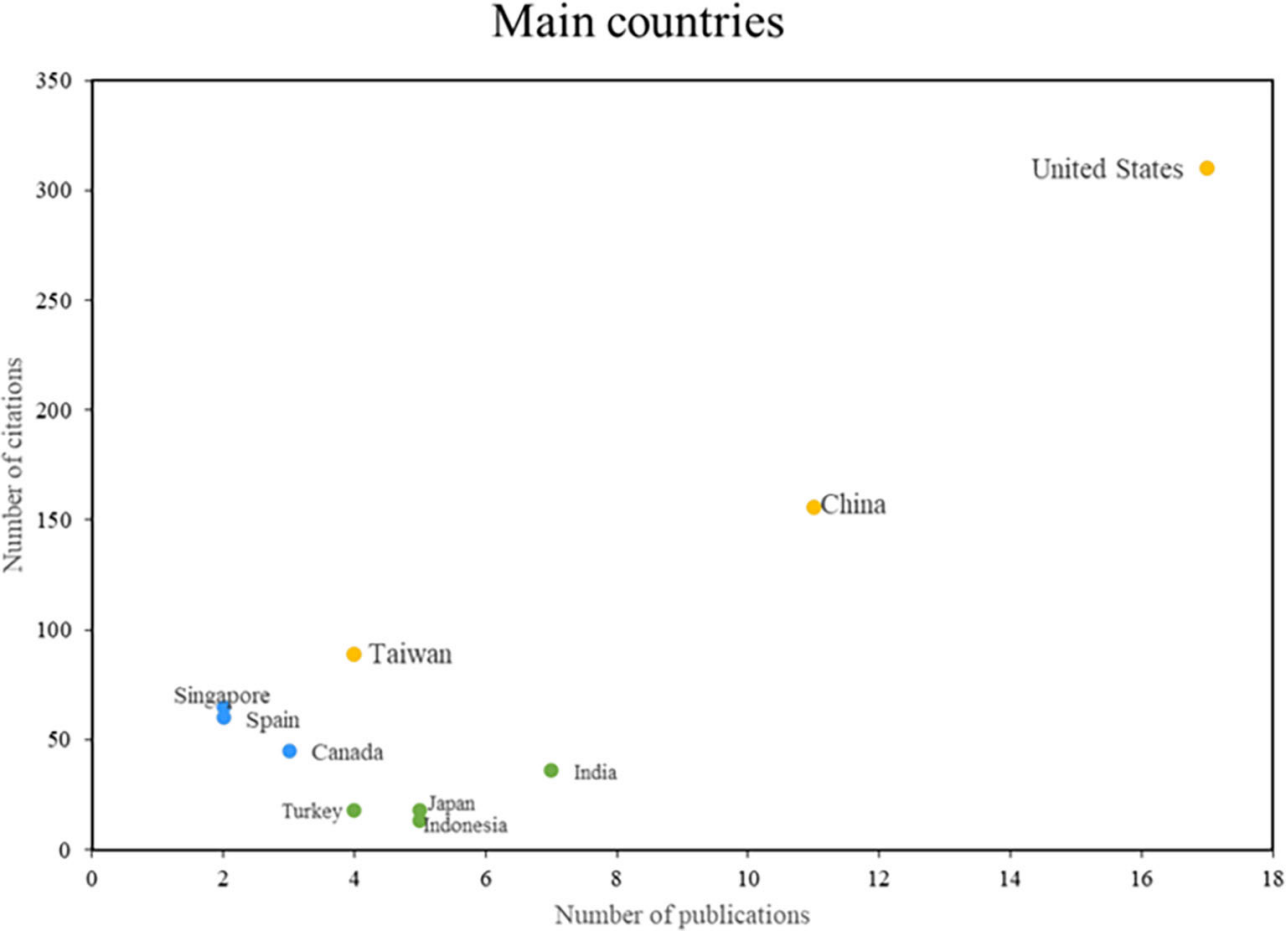
Main countries. Author's elaboration based on Scopus and Web of Science.

In the analysis of the main countries in this bibliometric study of smart restaurants, three groups can be distinguished as can be shown in Figure 5. First, some nations stand out in terms of productivity and scientific impact, such as the United States, Taiwan, and China, consolidating their positions as leaders in this field of research. On the other hand, there are countries such as Singapore, Spain, and Canada, which despite having a lower productivity index, are recognized for their importance and caliber. The impact of the third group of nations, including India, Japan, and Indonesia, is measured more by the volume of research published than by the number of citations. This places these countries in a position to actively contribute to the body of knowledge on smart restaurants. The country analysis provides a comprehensive view of the geographic distribution of research in this area.

**Figure 6.**
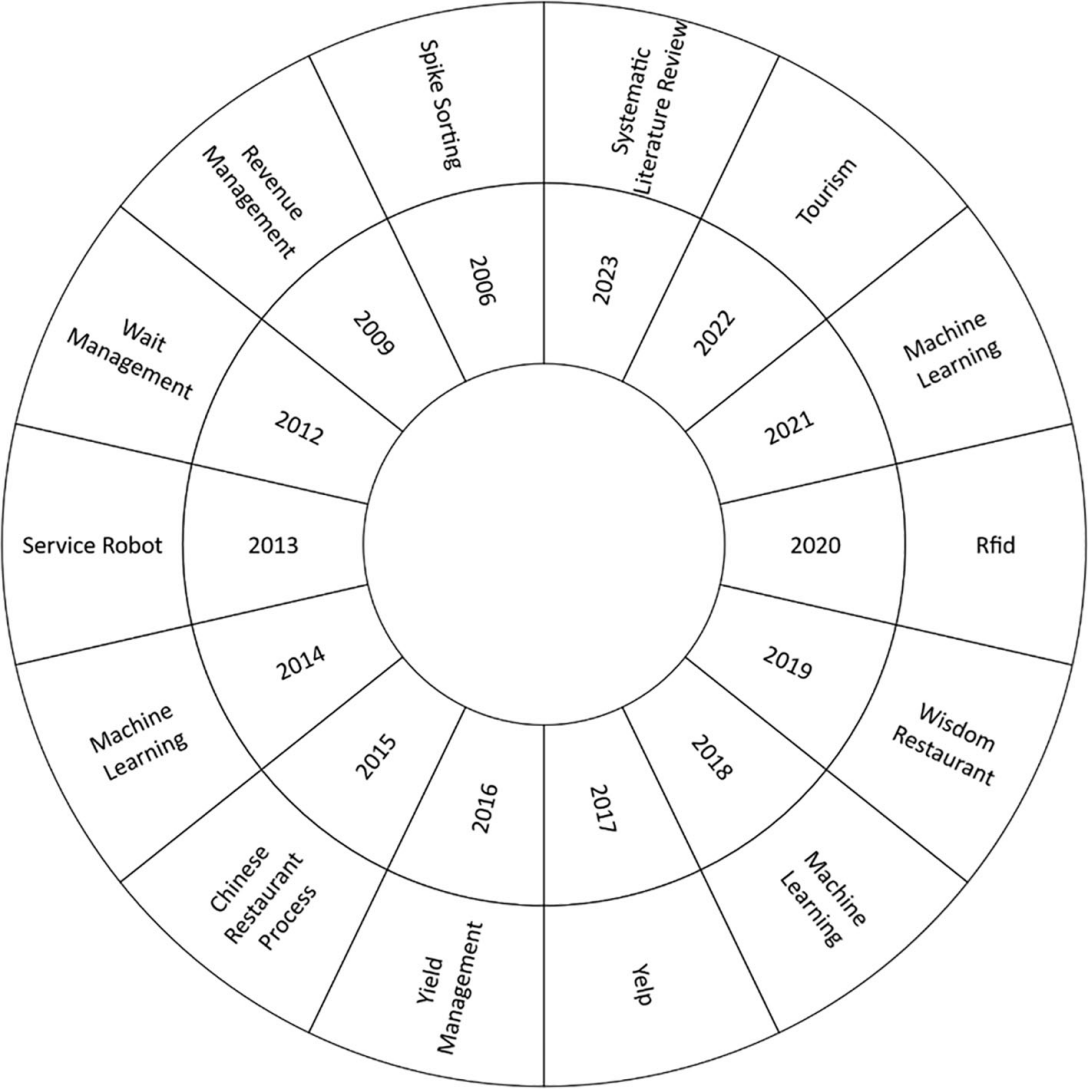
Thematic development. Own elaboration based on Scopus and Web of Science.

The current study, as shown in Figure 6, focused on the most used keyword in each research year to perform a comprehensive analysis of the thematic evolution in the Smart Restaurant literature between 2006 and 2023. As a starting point, it should be noted that 2006 is notable for the emergence of ideas such as “spike sorting.” Emerging topics such as “Radio-Frequency Identification” (RFID),” “Machine Learning,” “Tourism,” and “Systematic Literature Review" become more prevalent over the years, reflecting changing research dynamics and areas of interest. This thematic analysis provides valuable information on the current developments and trends in the field.

**Figure 7.**
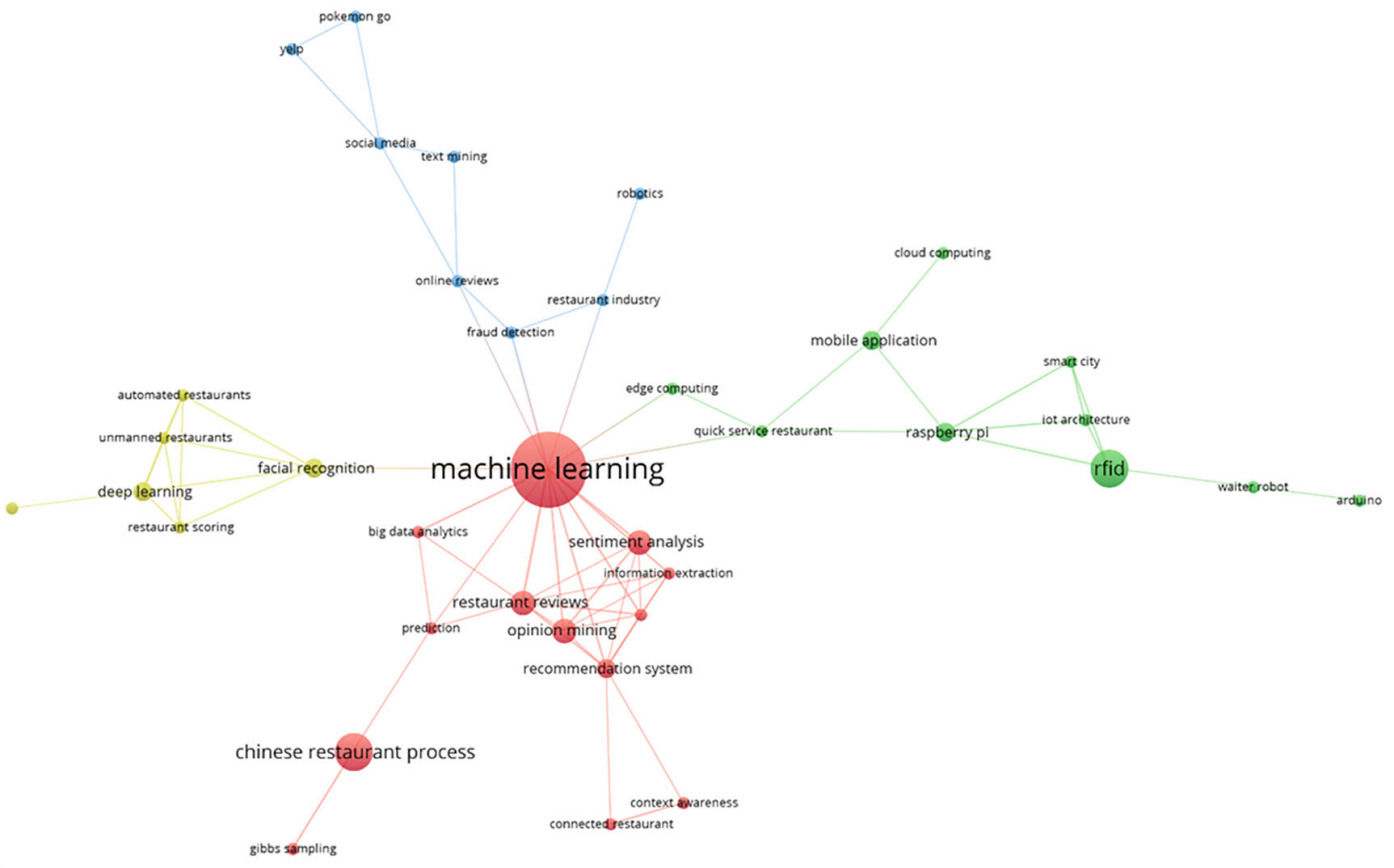
Keyword co-occurrence network. Own work based on Scopus and Web of Science.

The main keyword co-occurrence network is represented by these bibliometrics in four thematic groups, as shown in Figure 7. The red group, which includes terms such as “machine learning,” “big data analysis,” “opinion mining,” “Chinese restaurant process,” “prediction,” and “information extraction,” is the most prominent. The green group includes terms such as RFID, smart city, mobile application, edge computing, waiter robot, Arduino, and quick-service restaurant. A visual representation of the thematic interconnectedness in the scientific literature on this topic is provided by the identification of additional clusters in the area of smart restaurants, which are highlighted in blue and yellow, respectively. These groups capture the different aspects of conceptual affinity.

**Figure 8.**
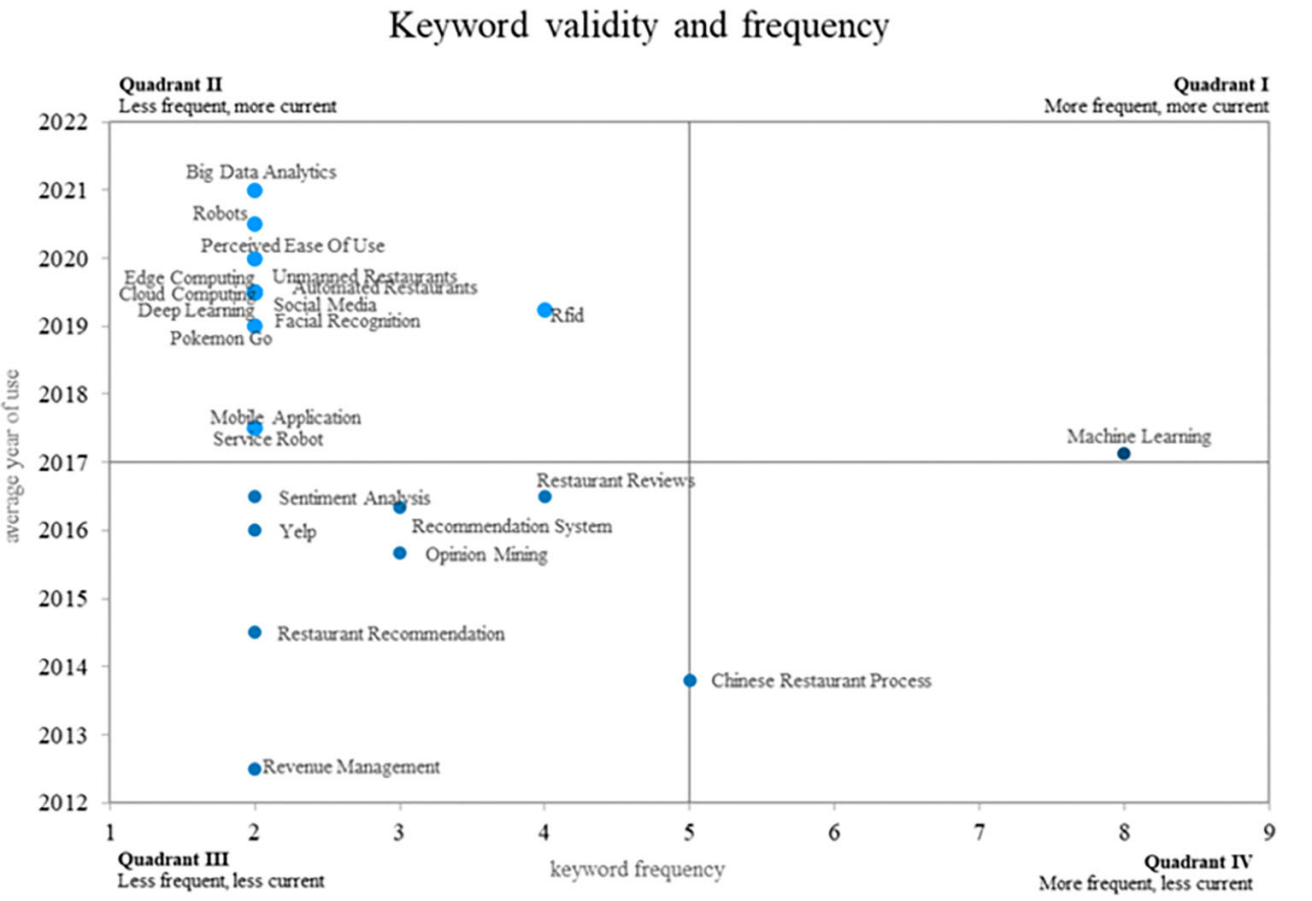
Validity and frequency of keywords. Own elaboration based on Scopus and Web of Science.

This bibliometric study of smart restaurants uses an innovative method to create four different quadrants using a Cartesian plane that combines the frequency of keyword use on the x-axis and the validity of that use on the y-axis. This method is illustrated in Figure 8. Quadrant 4 contains ideas that show a decreasing trend in use over time, as shown by terms such as “Chinese restaurant process. Quadrant 2 contains words that, although they appear infrequently, have great relevance in the recent literature, placing them in the category of emerging words. “Big data analytics, robots, edge computing, and cloud computing are some examples of these keywords. Last but not least, Quadrant 1 includes well-established and expanding ideas such as “machine learning,” which is still widely used and has applications in smart restaurant research. This method provides a distinctive visual representation of keyword dynamics in relevant scientific literature.

## Discussion

The Discussion section plays an essential role in the bibliometric analysis of smart restaurants. First, it thoroughly examines the research results, breaking down trends, patterns, and key findings. On the other hand, it provides a platform to explore the practical implications of these findings by identifying possible applications in the smart restaurant industry. Furthermore, it highlights the methodological limitations of the study, presents a classification of keywords according to their function in the literature, and highlights the main gaps in the research. Finally, it establishes a research agenda that identifies priority areas for future studies in the field of smart restaurants, thereby contributing to the understanding and continuous development of this topic.

### Analysis of the growth of the scientific literature on smart restaurants

In 2022, 2021, and 2018, a significant increase in scientific production related to smart restaurants was observed, with research addressing different aspects of this field with constant development. In 2022, research that analyzed the digital food environment was highlighted, providing a comprehensive view of how technology affects the food industry and its impact on the development of smart restaurants ( Granheim et al., 2022). Additionally, service quality in smart restaurants has been explored, identifying the relationship between “smart dining,” “smart restaurant,” and service quality (SSQ) ( Wong et al., 2022). These studies reflect the growing interest in understanding how technology reshapes customer experience and management in the restaurant industry.

In 2021, the impact of augmented reality on the customer experience in restaurants was examined, specifically in the case of “Le Petit Chef.” This study highlights how augmented reality technology is used to enhance culinary experience ( Batat, 2021). On the other hand, the potential of artificial intelligence and robotics in the restaurant sector in Europe was explored, assessing the potential for innovation in a highly interactive sector ( Blöcher & Alt, 2021). These studies highlight the growing integration of advanced technologies in the restoration sector and the need to understand their impact on the industry.

Similarly, in 2018, the focus was on food waste management in restaurants through the implementation of an Internet of Things (IoT) network. This study highlights how technology contributes to sustainability and efficiency in restaurant management ( Wen et al., 2018). In addition, warnings were issued regarding the arrival of automation in the hospitality industry, raising questions about the intrusion of robots into restaurant service delivery ( Bowen & Morosan, 2018). These studies anticipate the technological changes that occur in the hospitality industry and the need to adapt to these changes.

### Analysis of research references on smart restaurants

Zhang, Yoon, and Hwang have been recognized in the field of smart restaurants for their valuable contributions in several key areas. In particular, Zhang has noted his work on food waste management in restaurants, as seen in his study titled “Design, Implementation, and Evaluation of an Internet of Things (IoT) Network System for food waste management in restaurants.” ( Wen et al., 2018) This study demonstrated the effectiveness of implementing an IoT network for food waste management in restaurants, which is an essential aspect of sustainability and efficiency in the restaurant industry.

On the other hand, Yoon and Hwang have become research leaders for their studies on privacy and customer experience in restaurants. Their research, such as “Desired Privacy and the Impact of Crowding on Customer Emotions and Attraction-Avoidance Responses: Waiting in a Virtual Reality Restaurant” ( Hwang et al., 2012) and “Where Would You Like to Sit? Understanding customers’ privacy-seeking tendencies and location behaviors to create effective restaurant environments ( Hwang & Yoon, 2009) sheds light on how privacy and space arrangements in restaurants can influence emotions and customer responses. This research contributes to a better understanding of how to design effective restaurant environments and improve customer experience.

As for De Clercq, Wen, and Beck, their importance lies in their contribution to the aforementioned study, “Design, implementation, and evaluation of an Internet of Things (IoT) network system for food waste management in restaurants” ( Wen et al., 2018). Their participation was instrumental in addressing a significant problem in the restaurant industry and food waste management through the application of the IoT technology. Their innovative approach and search for practical solutions to restaurant management challenges have established them as leaders in this research field.

The Journal of Contemporary Hospitality and Waste Management has played an essential role in promoting the advancement of knowledge in the field of smart restaurants by publishing highly relevant and impactful research in this discipline.

The Journal of Contemporary Hospitality Management stands out for its contributions in various areas, such as customer experience management in smart restaurants. Studies such as “Desired Privacy and the Impact of Crowding on Customer Emotions and Approach-Avoidance Responses: Waiting in a Virtual Reality Restaurant” ( Hwang et al., 2012) have explored how privacy and the physical environment can influence customers’ emotions and responses. Similarly, “Artificial intelligence for big data analytics in hospitality: development of a prediction model for the usefulness of restaurant reviews for customer decision making” ( Lee et al., 2021) has addressed the application of artificial intelligence in data management for decision making in the restaurant industry, contributing to the advancement of data-based strategies in this sector.

On the other hand, Waste Management has been a key journal in promoting sustainable management in restaurants, especially with regard to food waste management. The study “Design, implementation and evaluation of an Internet of Things (IoT) network system for food waste management in restaurants” ( Wen et al., 2018) published in this journal has become a reference in the implementation of IoT technology to address the problem of food waste in restaurants. This study has had a significant impact on the restaurant industry by presenting innovative and sustainable solutions for waste management.

Both journals have contributed significantly to the advancement of research in the field of smart restaurants, and have allowed the dissemination of essential knowledge for the hospitality industry and restaurant management. Its influence continues in both the academic community and the professional practice of the field.

Research on smart restaurants has been led by different countries, each of which has made significant contributions to the advancement of the field. The United States has played an integral role in smart restaurant research, with notable studies such as “Beware Hospitality Industry: The Robots Are Coming,” ( Bowen & Morosan, 2018) which focuses on the automation and integration of robots in the hospitality industry, and “Learning user preferences with multiple information fusion for restaurant recommendations,” ( Fu et al., 2014) which focuses on learning user preferences for restaurant recommendations. These investigations have had a significant impact on the adoption of emerging technologies in restaurant management worldwide.

In Taiwan, the focus on customer experience in smart restaurants is noteworthy, as shown in “What Drives Experiential Loyalty Towards Smart Restaurants? The Case Study of KFC in Beijing,” (H. C. Wu & Cheng, 2018), which analyzes customer loyalty in smart restaurants, and “A deep learning facial recognition-based scoring system for restaurants” ( Chang et al., 2019) which uses facial recognition to assess customer experience. This research contributes to a deeper understanding of how smart restaurants can improve customer experience and foster loyalty.

China has emerged as a leader in smart restaurant research, with studies such as “smart dining, smart restaurants, and smart service quality (SSQ).” ( Wong et al., 2022) that focus on service quality in smart restaurants. In addition, “Learning Representations for Appearance Categories Detection in Online Reviews” ( Zhou et al., 2015) addresses the identification of appearance categories in online reviews, which is crucial for reputation management. restaurants. These studies have contributed significantly to the understanding of how technology can improve service quality in the restaurant industry.

In contrast, Singapore has excelled in research on sentiment analysis in smart restaurants, as reflected in “Vistanet: Visual Attention Network for Multimodal Sentiment Analysis” ( Truong & Lauw, 2019). This study focuses on multimodal sentiment analysis to understand customer opinions in smart restaurants. Spain has contributed with research such as “Grab, pay and eat: Semantic food detection for smart restaurants” ( Aguilar et al., 2018), which focuses on semantic food detection to improve the efficiency of order management in restaurants. Canada is also working on smart restaurant management, as seen in "Near-field communication sensors and cloud-based smart restaurant management system,” ( Saeed et al., 2016) which uses near-field communication sensors and the cloud to efficiently manage restaurants, and “Smart restaurants: Customer Demand Survey and Sales Forecasting” ( Lasek et al., 2016), which explores customer demand and sales forecasting in smart restaurants.

### Analysis of the thematic development of smart restaurants

The concept of “spike sorting” played a central role in the early years of smart restaurant research. This approach focuses on solving data management and information categorization challenges in restaurant systems ( Wood et al., 2006). Spike sorting enables the analysis and organization of critical data, which is valuable for managing real-time information in restaurant environments. As research progressed, the horizon expanded to include concepts such as RFID, machine learning, tourism, and systematic literature reviews, reflecting the need to address broader challenges and leverage emerging technologies to improve the efficiency and quality of intelligent restaurant management. This shift in conceptual focus underscores the adaptation of research to the changing needs of the restaurant industry, and highlights the importance of staying abreast of technological trends in the field.

Concepts such as RFID, machine learning, tourism, and systematic literature reviews have gained significant prominence in current research on smart restaurants. Radio Frequency Identification (RFID) technology has revolutionized inventory management and logistics in the restaurant industry, enabling the real-time tracking of products and improving operational efficiency. Meanwhile, machine learning is being used to personalize customer experience, from menu suggestions to reservation management, improving both customer satisfaction and restaurant efficiency ( Wong et al., 2022). Tourism and hospitality are important areas for smart restaurant research, as technological advancements are changing the way tourists interact with restaurant services at tourist destinations. Furthermore, the focus on a systematic literature review has allowed researchers to understand the current state of knowledge in the field of smart restaurants and identify critical gaps that require additional research ( Singh et al., 2023). These concepts have contributed to the growth and development of smart restaurant research, addressing fundamental questions and improving the efficiency and quality of service in the industry.

### Analysis of the smart restaurant topic clusters

The thematic cluster lists the most frequently used keywords and their relationships, as shown in Figure 7. Among these words, the main cluster was related to machine learning. This cluster is made up of terms such as: sentiment analysis, big data and analytics, restaurant reviews, opinion mining, recommendation system and prediction. These words usually constitute research oriented towards the use of predictive models built from data from previous reviews and opinions of consumers to facilitate decision making ( Hajek & Sahut, 2022) and provide options according to consumer preferences. users ( Lee et al., 2021). Also found in the same cluster are the terms: Chinese restaurant process, context awareness, connected restaurant, and Gibbs sampling.

The following cluster consists of the terms RFID, Raspberry Pi, Arduino, IoT architecture, smart cities, mobile applications, cloud computing, edge computing, and waiter robots. These terms form a cluster because of the use of RFID technology from the use of devices such as Raspberry and Arduino, which are integrated to form the IoT architecture and are part of the infrastructure of smart spaces, hosting the data in the cloud ( Harpanahalli et al., 2020; Wen et al., 2018). The following network of words comprises terms such as deep learning, facial recognition, restaurant automation, restaurant scoring, and unmanned restaurants, which are mainly part of the research that aims to automate restaurants using deep learning techniques based on restaurant scoring data ( Bonela et al., 2023; Dampage et al., 2021). The last cluster consists of the terms fraud detection, restaurant industry, robotics, online reviews, text mining, social media, Yelp and Pokemon Go.

### Analysis of frequency and conceptual validity around smart restaurants

The concept of “Chinese Restaurant Process” (CRP) was located in quadrant 4 of the Cartesian plane, indicating that its frequency of use has decreased compared to previous periods in smart restaurant research. CRP is a statistical technique used in data modeling, particularly in the fields of artificial intelligence and machine learning. Historically, CRP has been applied in contexts such as neural signal processing and decision making in dynamic environments ( Wood et al., 2006). It has also been used to improve reinforcement learning algorithms ( Shi et al., 2018), where it is used in the context of reinforcement learning to improve decision-making efficiency.

However, as research on smart restaurants has progressed, CRP may become less relevant in this specific context. The decline in its use may reflect changes in research trends and an increased focus on other concepts more relevant to the smart restaurant industry, such as RFID, machine learning, and others mentioned above. This suggests that smart restaurant researchers are exploring new directions and adapting their approaches to meet technological and industrial needs.

Quadrant 2 of the Cartesian plane, which houses novel concepts in the scientific field of smart restaurants, reflects the growing importance of terms such as big-data analytics, robots, edge computing, and cloud computing in the present and near future. These terms are indicative of technological trends and the need to adapt to ever-changing business environments.

The practice of big data analytics has established itself as a fundamental pillar in restaurant management, enabling the collection and analysis of large amounts of data with the aim of obtaining valuable information about customer preferences and supporting decision-making ( Lee et al., 2021). On the other hand, the integration of robots in restaurants is revolutionizing the industry by automating tasks such as food and customer services, helping to improve both efficiency and customer experience ( Akhund et al., 2020).

Edge and cloud computing play essential roles in facilitating real-time connectivity and data processing in smart restaurants ( Aytaç & Korçak, 2018). These technologies enable rapid data transfer between Internet of Things (IoT) devices and central systems, which is critical for efficient restaurant management ( Saeed et al., 2016).

Quadrant 1 of the Cartesian plane, where concepts are constantly growing and occupying a central and consolidated place in smart restaurant research, highlights the current and future importance of machine learning. Machine learning, also known as automatic learning, has played a fundamental role in restaurant management, allowing companies to analyze large datasets and extract valuable information to support strategic decision-making. This study illustrates how Machine Learning has been used to analyze restaurant review data and to develop predictive models to help customers make decisions ( Lee et al., 2021).

In addition, machine learning has been successfully applied to fraud detection in the restaurant industry ( Hines & Youssef, 2018), where it is used to identify rotating check fraud. This technology is essential for identifying unusual patterns and for preventing potential financial losses.

### Classification of keywords related to smart restaurants according to their functions

Table 1 presents a classification of emerging and growing keywords related to the development of smart restaurants, according to their functions. This allows the identification of the main characteristics and applications associated with each category, highlighting constantly growing areas of interest and prominent approaches in current research.

**Table 1.** Classification of keywords according to their function. Own elaboration based on Scopus and Web of Science.

Keyword	Associated Tools	Applications	Characteristics
Machine Learning	Big Data Analytics	Restaurant Review	Leverage machine learning from user review data on the web and social networks for predictive modeling and decision making
		Restaurant reviews	
		Information Extraction	
		Sentiment Analysis	
		Text mining	
Restaurant automation	Deep Learning	Facial Recognition	Using deep learning techniques to program robotic waiters to assist diners, automate processes, and improve service times.
		Waiter robots	
		Fast service in restaurant	
IoT Architecture	Raspberry PI	Restaurant Industry	Use programmable devices and RFID technology to implement an IoT architecture to launch the smart restaurant, based on the provision of data in the cloud and the generation of predictive models.
	RFID		
	Arduino		
	Cloud Computing		

### Theoretical implications

Conducting bibliometrics on smart restaurants with a focus on different dimensions, such as the frequency of publications over the years, identification of prominent theoretical references, monitoring of thematic evolution, analysis of the co-occurrence of keywords, exploration of emerging and growing keywords, and identification of unexplored areas of research, has important theoretical implications for the advancement and understanding of this field of study.

First, analyzing the frequency of publications over the years provides an overview of the dynamics of research over time. This allowed us to identify periods of greater research activity, thus revealing the relevance and interest in the topic at different times. The identification of key years, such as 2022, 2021, and 2018, with a high number of publications suggests the importance of these periods for the development of smart restaurant research.

Identification of the main theoretical references in bibliometrics provides a deeper understanding of the theoretical and methodological approaches that have guided research in this field. Authors such as Zhang, Yoon, Hwang, De Clercq, Wen, and Beck stand out in terms of productivity and impact, indicating their significant contribution to the existing body of knowledge.

Furthermore, the analysis of thematic evolution in bibliometrics reflects how smart restaurant research evolved from initial concepts such as “spike sorting” in the early years to emerging and consolidated areas such as “machine learning,” RFID, “edge computing,” and “big data analytics.” This analysis not only helps to understand the historical trajectory of research but also identifies current and future trends in the field.

Keyword co-occurrence analysis reveals the conceptual structure of the research field and how key terms are related. The identification of thematic clusters and highlighted keywords within each cluster provided an overview of the most relevant areas of focus in smart restaurants.

The analysis of emerging and growing keywords allows for the identification of concepts that are gaining importance and are expected to have a significant impact on future research. Terms such as “robots,” “edge computing,” and “cloud computing” emerge as areas of emerging interest

suggesting new directions for research

Finally, the identification of unexplored research areas is a crucial implication of bibliometrics. By analyzing the gaps in the existing literature, valuable guidance is provided to researchers and academics interested in addressing underexplored areas or identifying unresolved research questions in the field of smart restaurants. These gaps can serve as a starting point for future research and contribute to the theoretical and practical advancement of the discipline.

### Practical implications

Conducting bibliometrics on smart restaurants with a focus on thematic evolution and keyword analysis has practical implications of great relevance for both the restaurant industry and academic community. The transition from research focused on the concept of spike sorting to emerging areas, such as RFID, machine learning, tourism, and systematic literature review, reflects a growing understanding of the importance of technology and data management in improving efficiency and quality in the restaurant industry. This means that industry professionals must stay abreast of emerging trends and consider implementing technologies, such as machine learning and big data analytics, to optimize decision making and improve customer experience.

The identification of thematic clusters and the conceptual relationship between key terms, such as machine learning, big data analytics, and restaurant reviews, highlight the importance of interdisciplinary research and collaboration between researchers and professionals from different fields. This suggests that smart restaurants can benefit from a comprehensive approach that integrates advanced technologies with a deep understanding of customer preferences and opinions, which in turn can influence strategic decision-making and service quality management.

Analyzing keyword frequency and relevance provides valuable insights into current trends in the industry. The emergence of concepts such as big data analytics, robots, edge computing, and cloud computing underscores the importance of digitization and automation in the restaurant industry. This suggests that smart restaurants can improve their operational efficiency and service quality by implementing advanced technologies such as robots and cloud-based management.

However, the continuous increase in the frequency of keywords related to Machine Learning indicates that this approach remains relevant and crucial in the research and application of technologies in smart restaurants. The practical implications of this finding are significant as they highlight the importance of training and adopting machine learning models for a variety of applications, such as service personalization and customer data management.

In addition, bibliometrics provide insights into best practices in research and methodology in the field of smart restaurants. This knowledge can be used by researchers and academics to understand the most effective techniques and approaches for generating knowledge in this area. Likewise, the analysis of thematic development can serve as a guide to identifying areas of research that have received disproportionate attention or, on the contrary, have been insufficiently addressed, which can improve the quality and relevance of future research projects.

Another important practical implication is related to education and training in hospitality and restaurant management. The results of bibliometrics can be used to develop education and training programs to prepare students and professionals to meet the changing technological demands of the food and hospitality industry. Emerging and growing concepts, such as machine learning or big data analytics, can be incorporated into curricula to ensure that future professionals acquire the necessary skills.

The research offers practical guidance for restaurant managers and owners to effectively adopt emerging technologies in their operations. By identifying key trends and areas of focus, industry professionals can make informed decisions about implementing technological solutions that improve operational efficiency and service quality. Keyword analysis highlights the importance of interdisciplinary collaboration and integrating technology with a deep understanding of customer preferences and opinions. This underscores the need for strategic management that combines technical knowledge with a customer-focused vision to enhance the dining experience and increase loyalty.

The research has a significant impact on the food and beverage industry by providing a roadmap for innovation and continuous improvement. It offers detailed insights into trends and best practices in the smart restaurant space, helping professionals keep up with technological developments and changing consumer demands. This benefits restaurant owners by improving their competitiveness and profitability, while also contributing to customer satisfaction and the sustainable growth of the industry as a whole.

The research emphasizes the significance of hotel and restaurant management training and education from a societal perspective. This is to prepare the next generation of professionals to meet the industry’s technological challenges by integrating emerging technological concepts and tools into curricula. Educational institutions can ensure that students acquire the skills necessary to thrive in an increasingly digitalized business environment. Encouraging the adoption of technologies that improve efficiency and quality of service can contribute to the creation of employment and economic development in the restaurant sector.

### Limitations

The present bibliometrics on smart restaurants, carried out using the PRISMA-2020 methodology and based on the Scopus and Web of Science databases, provides a valuable overview of research in the field. However, as is usually the case with bibliometric studies, it has certain limitations that must be considered when interpreting results. First, it is important to emphasize that the quality and completeness of the databases used may have affected the representativeness of the results. Despite efforts to cover a wide range of academic publications, some relevant research may have been absent or underrepresented in the selected databases. In addition, inherent limitations in keywords and search criteria may have influenced the selection of articles for inclusion in the analysis, which could have affected the breadth and depth of the identified research landscape.

Another limitation is related to the quality of the bibliographic data available in the databases. Errors in the cataloging of publications, lack of uniformity in the introduction of metadata, and duplication of records may have influenced the precision of the bibliometric indicators used in this study. Furthermore, differences in how publications are cited in different disciplines and contexts may have influenced keyword co-occurrence analysis and the identification of thematic trends.

Finally, it is important to recognize that while bibliometrics provide a valuable overview of the scientific production in a field, they do not have the capacity to fully capture the quality, relevance, or real impact of research. The sheer number of publications does not always reflect the depth or influence of a field of study, and it is essential to complement bibliometric findings with qualitative assessments and critical literature reviews to obtain a complete understanding of the current state of smart restaurant research.

### Research gaps

Table 2 summarizes the main underexplored research areas identified in the bibliometric study of smart restaurants. These areas represent areas where future research could focus on advancing our knowledge. Highlighted areas of need include further exploration of the implementation of emerging technologies, such as artificial intelligence and the Internet of Things, in restaurant management and operations. The importance of exploring how digital experiences and customer interactions reshape the restaurant industry and how they can be optimized is also highlighted.

**Table 2.** Research gaps. Author's calculations based on Scopus and Web of Science.

Category	Investigative Gaps	Justification of the Gap	Questions for Future Researchers
Thematic Gaps	Integrating sustainability into smart restaurants	Sustainability is a key issue in the food industry, but more research is needed on how technologies in smart restaurants can contribute to economic and environmental sustainability.	How can intelligent restaurant technologies promote sustainable practices? What are the implications for economic and environmental sustainability?
	Social and Cultural Effects of Technologies in Restaurants	Despite their adoption, there is little understanding of how smart restaurant technologies affect social interactions and the perception of the dining experience.	How do technologies influence the customer experience and the authenticity of the dining experience?
Geographic Gaps	Regional context and adaptation of technologies in developing countries	Most research focuses on developed countries, leaving a gap in understanding how these technologies are applied in regional contexts and in developing countries.	How are technologies implemented in smart restaurants in regional contexts and developing countries? What are the unique challenges in these regions?
Interdisciplinary Gaps	Multidisciplinary collaboration to research smart restaurants	Because this is an interdisciplinary field, collaboration between experts in technology, sustainability, and experience design is essential, but currently limited.	How can experts from different disciplines collaborate effectively to address smart restaurant challenges? What are the advantages of a multidisciplinary perspective?
Temporal Gaps	Long-Term Evolution of Smart Restaurants	Given the rapid evolution of this field, research is needed to understand how it will evolve in the long term and which trends will prevail.	What are the expected long-term trends in smart restaurants? How will these technologies evolve in the coming years?

Other areas highlighted in the table include the lack of research specifically dedicated to sustainability in smart restaurants as well as the lack of studies that take a detailed look at data management and cybersecurity in this context. The influence of cultural and regional factors on the adoption of smart technologies in restaurants is another area that is considered relevant for future research.

### Research agenda

**Figure 9.**
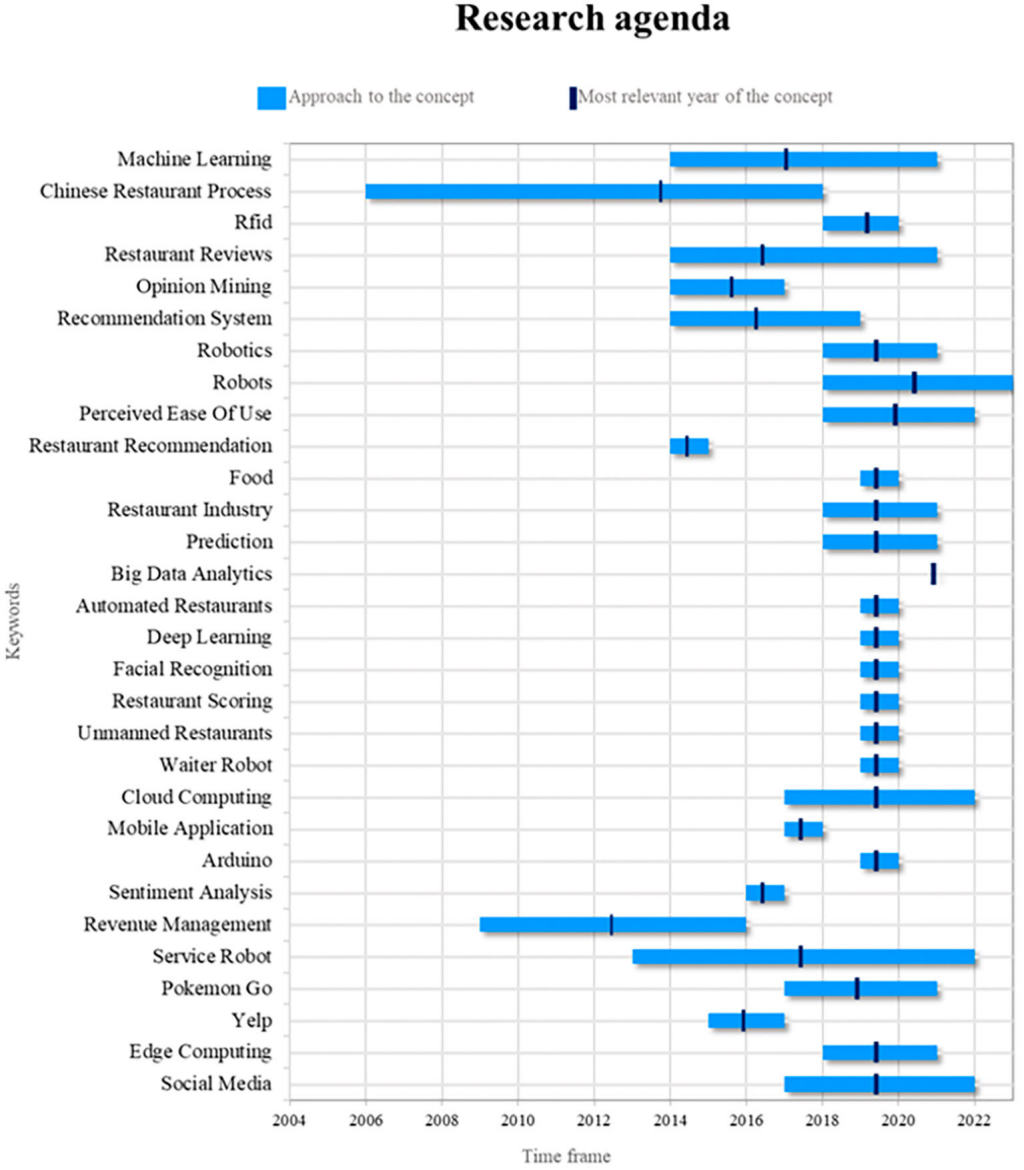
Research agenda. Author's elaboration based on Scopus and Web of Science.

Robots are essential components of the smart restaurant industry. Their relevance lies in their ability to automate repetitive tasks, improve operational efficiency, and provide customers with unique experiences. In future research, it will be possible to explore more advanced approaches to programming robots for specific tasks, such as food delivery and interaction with diners. In addition, research can be conducted on how artificial intelligence and machine learning can improve the ability of robots to adapt to changing restaurant environments, including the ability to recognize and respond in a more personalized manner to customer preferences (see Figure 9).

Another interesting aspect that could be the subject of future research is the ethics of robotics in restaurants. Addressing issues related to the privacy and security of data collected by robots as well as the impact on the quality of employment in the service industry are areas that deserve further analysis. In addition, investigating how restaurants can ensure customer comfort and acceptance of robots in their establishments is critical for promoting their adoption and ensuring their continued success.

The concept of the Chinese Restaurant Process (CRP) has been central to smart restaurant research, but its relevance has waned in recent years. However, this still offers significant potential for future research. Potential applications of CRP can be explored in resource allocation and inventory management in restaurants, with the goal of improving operational efficiency and reducing food waste. In addition, CRP can have significant implications in analyzing customer preferences and customizing menus based on order history.

On the other hand, the concept of “service robots” has become increasingly important in the context of smart restaurants. Future research could focus on how to effectively integrate service robots with CRP to improve both the customer experience and operational efficiency. In addition, restaurant review analysis is critical for decision making and customer feedback. Future studies could investigate how CRP can contribute to the automated analysis of restaurant reviews, and how to improve service quality based on customer feedback. These investigations have the potential to revitalize the relevance of CRP in the context of smart restaurants and contribute to the advancement of the industry.

## Conclusions

After analyzing the bibliometrics of smart restaurants, it is clear that interest and research in this area has experienced a significant increase in recent years. In particular, 2021 and 2022 have emerged as the most productive years in terms of scientific production on the topic. This increase is not linear; however, the number of scientific articles has grown exponentially by an impressive 95.36%. This dynamic demonstrates strong academic and professional enthusiasm for further understanding and developing this emerging industry.

In terms of the main references of the research, Zhang H, Yoon S-Y and Hwang J stand out as the most influential authors in the field, with the International Journal of Contemporary Hospitality Management serving as the main vehicle for the dissemination of these studies. It is also interesting to note the geographical concentration of scientific production, with the United States, China, and Taiwan being pioneering countries in research on smart restaurants.

At the thematic level, there has been evolution in the topics studied. In the beginning, research focused on more technical aspects such as spike sorting; today, it has diversified to cover areas such as tourism and systematic literature review. However, the main thematic clusters highlight the incorporation of artificial intelligence and data analysis techniques into the sector, with key concepts such as machine learning, sentiment analysis, and recommendation systems leading the discussion.

The field is constantly evolving, as evidenced by the emergence of keywords. Consolidated concepts, such as machine learning, coexist with growing terms, such as big data analytics, robots, perceived ease of use, and edge computing. These terms not only highlight current trends but also suggest possible directions for future research.

Finally, in the future, the smart restaurant research agenda should focus on deepening these emerging and consolidated concepts. Given the rapid evolution of this field, it is essential to maintain an up-to-date perspective and be prepared to address the challenges and opportunities that these technological and thematic changes will bring to the design and operation of restaurants.

## Ethics and consent

Ethical approval and consent were not required.

Reporting guidelines: PRISMA checklist DOI: https://doi.org/10.5281/zenodo.14146154 ( Valencia Arias and Cardona Acevedo, 2024).

Data are available under the terms of the Creative Commons Attribution 4.0 International license (CC-BY 4.0).

## Data Availability

The data availability statement for this study has been duly registered and archived in the Zenodo open data repository, which is recognized for its commitment to the accessibility and preservation of scientific data. The data and materials supported by this study are publicly available under a Creative Commons Attribution 4.0 International (CC BY 4.0) license and can be accessed at the following DOI link: https://doi.org/10.5281/zenodo.14146154. ( Valencia Arias and Cardona Acevedo, 2024).
