# Trends in Smart Restaurant Research: Bibliometric Review and Research Agenda

**DOI:** 10.12688/f1000research.158066.2

**Published:** 2025-02-17

**Authors:** Alejandro Valencia-Arias, Sebastián Cardona-Acevedo, Ezequiel Martínez Rojas, Juana Ramírez Dávila, Paula Rodriguez-Correa, Lucia Palacios-Moya, Renata Teodori de la Puente, Erica Agudelo-Ceballos, Martha Benjumea-Arias

**Affiliations:** 1 School of Industrial Engineering, Universidad Senor de Sipan, Chiclayo, Lambayeque, 14001, Peru; 2 Centro de Investigaciones Escolme-CIES, Institución Universitaria Escolme, Medellín, 050005, Colombia; 3 Vicerrectoría de Investigación e Innovación, Universidad Arturo Prat, Iquique, Tarapacá Region, Chile; 4 Departamento Estudios Generales, Universidad Senor de Sipan, Chiclayo, Lambayeque, Peru; 5 Escuela de posgrado, Universidad Senor de Sipan, Chiclayo, Lambayeque, Peru; 6 Instituto de Investigación y Estudios de la Mujer, Universidad Ricardo Palma, Santiago de Surco, Peru; 7 Departamento de Ciencias Administrativas, Instituto Tecnológico Metropolitano, Medellín, Colombia

**Keywords:** Smart restaurants; PRISMA; machine learning; deep learning; IoT.

## Abstract

**Background:**

The automation of processes and services has transformed various industries, including the restaurant sector. Technologies such as the Internet of Things (IoT), machine learning, Radio Frequency Identification (RFID), and big data have been increasingly adopted to enhance service delivery, improve user experiences, and enable data traceability. By collecting user feedback and analyzing sentiments, these technologies facilitate decision-making and offer predictive insights into future food preferences. This study aims to explore current research trends in intelligent restaurants, focusing on technological applications that improve service and decision-making.

**Methods:**

A bibliometric analysis was conducted in accordance with the PRISMA-2020 guidelines. A total of 94 academic documents were reviewed from the Scopus and Web of Science databases, focusing on publications related to intelligent restaurant systems, particularly involving IoT and automation technologies.

**Results:**

The analysis revealed that the United States, India, and China have contributed the most to the field, with a particular emphasis on China’s implementation of IoT architecture and robotics in restaurant settings. Chinese restaurant innovations, particularly in robotics, are among the most frequently cited in the literature. The study identifies these countries as leading the research in the intelligent restaurant domain.

**Conclusions:**

Technologies such as IoT, machine learning, RFID, and big data are driving advancements in restaurant automation, enhancing service efficiency and user experience. The United States, India, and China are leading research in this area, with China standing out for its application of robotics and IoT in restaurants. This research provides a foundation for future studies aimed at improving predictive models for food selection and service optimization.

## Introduction

The intensive use of various advanced technological tools in different economic sectors is becoming increasingly widespread. The development of information and communication technologies (ICT) has brought about significant changes in the way people interact and adapt to different social environments ( Bai & Wu, 2022). Thus, the maturity of technology has influenced the productivity and daily life of people who use these tools to have a better quality of life, greater efficiency in their daily activities, and to enjoy the convenience they have brought ( Bai & Wu, 2022).

Tools such as Artificial Intelligence (AI), the Internet of Things (IoT), building information, big data, cloud computing, machine learning ( Wu & Huang, 2020), and automation tools have allowed companies dedicated to different activities to achieve better results in terms of productivity, time, competitiveness, and economics ( Jubaid et al., 2023). The adoption of these technologies makes it possible to create new types of business architectures and build sustainable and intelligent industries ( Elkhwesky & Elkhwesky, 2023). One sector in which the adoption of technological tools has expanded is the tourism and services sector, especially in restaurants ( Singh et al., 2023).

In this sense, as users become more comfortable with technologies that allow, for example, self-service, automatic ordering and payment systems, and robotic services, this type of technology will continue to become more relevant in restaurants ( Wong et al., 2022). Some of these technologies became more relevant, especially in the context of Covid-19, where one of the most affected industries was restaurants, which, in order to adapt to the new reality, were forced to implement this type of technology to provide their services again ( Hasan et al., 2022).

However, some restaurants have continued to implement technologies to improve the user experience and update the way they provide the service. Some of the technologies that have been adapted include a personal digital assistant (PDA), Wi-Fi access, and high-end monitors for menu selection, including the use of holograms ( Kavitha et al., 2022). To adapt to the context of smart cities, restaurants are transforming into smart restaurants. For example, the use of augmented reality in menus can enhance the user’s dining experience and facilitate operations related to ordering and billing by automating this entire process ( Prathibanandhi et al., 2022).

The integration of elements such as cloud computing allows different services to be hosted in the cloud and has information available to customers and employees; the use of sensors and IoT devices allows restaurants to have better management of the restaurant, providing in-formation on the occupancy of the premises, interactive menus, availability of food, process and verification of food quality, and organization ( Martino et al., 2022). One of the advances that has had the greatest impact on restaurants is the use of robots for user service, either as waiters or just to take the order ( Mishra et al., 2022). However, the use of this type of technology requires the integration of different tools, such as Internet access services, control, monitoring, and identification services, to integrate people, things, and objects ( Wu & Huang, 2020).

In addition, it is possible to create interactive menus that can identify user preferences based on data collection, and through the use of chatbots, it is possible to collect purchase data and provide quick responses to consumers by making personalized recommendations ( Kaur et al., 2023).

However, in restaurants, robots need to understand the environment and make decisions accordingly, such as changing their route if they encounter obstacles along the way. To have the ability to make decisions and behave like humans, robots need information from multiple sources from which they can understand the change in the environment, the position of other robots, and the path to the destination. This can be achieved by integrating robots with IoT technology, which improves the capabilities of a robotic waiter ( Mishra et al., 2022).

The above not only allows for a personalized and more interactive experience for the user but also increases the possibility of meeting their expectations. This is because today’s users are much more demanding, have more options to choose from, and have more bargaining power in addition to being content generators who share their experiences with other users ( Almeida et al., 2023). On the other hand, it is also possible to support marketing strategies through the use of digital tools, allowing the restaurant’s competitive advantage to be improved using data from reviews on social networks, patterns of feelings, and behavior through the use of big data ( Hajek & Sahut, 2022).

The adoption of technology within the restaurant sector remains comparatively low due to numerous interconnected factors. Primarily, a notable impediment is the reluctance of restaurant owners and managers to adopt change, as many adhere to conventional practices. Concerns regarding the financial implications of investment, the complexity of implementation, and the potential disruption to daily operations further contribute to this reluctance. Additionally, a dearth of knowledge and understanding regarding the effective integration of technology into restaurant operations engenders uncertainty, which in turn leads to the avoidance of adoption. The internal environmental locus of control—defined as the perception of control over a restaurant’s internal environment—has been identified as a pivotal factor in determining behavioral intentions toward technology adoption. This underscores the importance of addressing the attitudes and perceptions of owners, managers, and staff to facilitate smoother integration of technological advancements ( Joo, Kim, & Hwang, 2023).

Furthermore, the dearth of access to requisite resources and training further curtails technology adoption in the restaurant sector. Many owners and managers may lack the technical expertise necessary to assess, select, and implement technological solutions that are optimally suited for their operations. Furthermore, inadequate technological infrastructure, such as unreliable internet connectivity, can impede the effective implementation of advanced technologies. The role of consumer attitudes and loyalty towards smart vending technology in restaurants is also crucial, with customer perceptions and experiences influencing the decisions of restaurant operators ( Lacap et al., 2023). These factors, both perceived and real, can contribute to the low adoption rates of technology in the restaurant sector.

While the adoption of similar technologies has been widely studied in other sectors, the hospitality and tourism industry, including restaurants, still requires more attention. The adoption of technology remains relatively low in this field ( Elkhwesky & Elkhwesky, 2023; Klouvidaki et al., 2023). Consequently, further research is necessary to identify the key factors and advances that can facilitate the integration of technological elements in the development of smart restaurants. The objective of this study is to systematically analyze current research trends in the field of smart restaurants to identify areas that necessitate further exploration. A comprehensive bibliometric review of existing literature will be employed to provide a global perspective on prevailing approaches in the development of smart restaurants and to inform a research agenda for future studies. By gaining a more profound understanding of customer needs and preferences, as well as the challenges and benefits associated with the implementation of emerging technologies, this research seeks to offer clear guidance for strategic decision-making and planning in the restaurant sector.

Moreover, the objective of this study is to make a theoretical contribution to the field of innovation and technology management in the hospitality industry. The research will address specific challenges faced by restaurants in the digital era by identifying relevant models and conceptual frameworks. The findings of this research are expected to provide valuable insights for restaurant owners to improve their operations and services. Moreover, the findings may contribute to the advancement of the food and beverage industry by fostering innovation and the adoption of technologies that enhance competitiveness and long-term sustainability. The practical and strategic insights derived from this research are poised to assist restaurants in adapting to a dynamic environment, enhancing their competitiveness, and addressing the escalating expectations of customers in the digital age.

RQ1: What are the years of greatest interest in research on smart restaurants?

RQ2: What kind of growth exists in the number of scientific articles about research on smart restaurants?

RQ3: What are the main research references on smart restaurants?

RQ4: What is the thematic evolution of scientific production in smart restaurants?

RQ5: What are the main thematic clusters on smart restaurants?

RQ6: What are the growing and emerging keywords in Smart Restaurants research?

RQ7: Which topics are positioned as protagonists in the design of a research agenda on smart restaurants?

In this sense, the article is composed of a methodological section, which details the way the article is developed, as well as the sources of information, the results or findings obtained, their discussion, as well as the identification of research gaps, the approach to the research agenda, and, finally, the main conclusions.

## Methods

As part of this exploratory study on smart restaurants, a bibliometric analysis was conducted using the guidelines described in the PRISMA-2020 statement. ( Page et al., 2021; Valencia-Arias et al., 2024; García-Pineda et al., 2024). The collection and review of relevant secondary research materials forms the basis of this methodology.

### Eligibility criteria

The establishment of inclusion criteria is imperative for the selection of relevant texts for analysis in this bibliometric study on Smart Restaurants. To ensure the quality and relevance of the documents considered, those that did not directly contribute to the field of study were excluded. This procedure enabled the bibliometric analysis to concentrate exclusively on research that is aligned with the objectives of the study, thereby ensuring the validity of the results obtained.

The document selection and exclusion processes were meticulously executed in three phases. In the initial phase, records that were duplicates or contained indexing errors in the Scopus and Web of Science databases were eliminated. In the subsequent phase, studies lacking complete access to the full text were excluded, as a bibliometric review necessitates comprehensive information for an accurate analysis of trends and co-occurrences. In the final phase, documents were filtered according to their thematic relevance, with the objective of including only those that directly addressed research on smart restaurants and applied technologies. This methodological approach ensures that the analyzed database accurately reflects the trends and development of the literature in the field.

### Source of information

To ensure the quality and relevance of the documents analyzed, the Web of Science and Scopus databases were selected, as they are widely recognized in bibliometric studies for their rigour, coverage, and indexing quality. While alternative databases, such as Google Scholar, IEEE Xplore, or specialized databases, might contain additional publications, the utilization of Web of Science and Scopus aligns with the objective of ensuring that the studies originate from high-quality, peer-reviewed sources, thereby circumventing biases stemming from unverified literature. This decision is further substantiated by prior studies that have validated the exclusive use of these databases in bibliometric analysis within applied technologies and service sectors. The legitimacy of these databases as the primary sources of academic and scientific information is further substantiated by their comprehensive coverage, encompassing a vast array of disciplines and providing access to numerous academic journals and conferences. This assertion is corroborated by a comparative study of Scopus and Web of Science in a typical university environment, which underscores their value and effectiveness in searching and retrieving scientific literature ( Vieira & Gomes, 2009).

### Search strategy

To perform an efficient search, two specialized search equations were developed for the two selected databases, Scopus and Web of Science. These equations were carefully designed to meet the previously established inclusion criteria while also being tailored to the unique search requirements of each database. This strategy allowed them to retrieve relevant scientific literature and ensure the accuracy and integrity of the data collection for ongoing bibliometric research.

For the Scopus database: (TITLE (“Smart Restaurant*” OR “smart restaurant*" OR (iot AND restaurant*) OR (“internet of things” AND restaurant*) OR (“artificial intelligence” AND restaurant*) OR (“virtual reality” AND restaurant*) OR (“augmented reality” AND restaurant*)) OR KEY (“Smart restaurant*” OR “smart restaurant*”) OR (iot AND restaurant*) OR (“Internet of Things” AND restaurant*) OR (“Artificial Intelligence” AND restaurant*) OR (“Virtual Reality” AND restaurant*) OR (“Augmented Reality” AND restaurant*))).

For the Web of Science database: (TI=(“intelligent restaurant*” OR “smart restaurant*" OR (iot AND restaurant*) OR (“Internet of things” AND restaurant*) OR (“Artificial intelligence” AND restaurant*) OR (“Virtual reality” AND restaurant*) OR (“Augmented reality” AND restaurant*)) OR AK=(“Smart restaurant*” OR “intelligent restaurant*" OR (“IoT AND restaurant*”) OR (“Internet of Things AND restaurant*”) OR (“Artificial Intelligence AND restaurant*”) OR (“Virtual Reality AND restaurant*”) OR (“Augmented Reality AND restaurant*”))).

Each search equation was meticulously engineered to align with the distinctive characteristics and requirements of the Scopus and Web of Science databases, taking into account their varied search logics. In the case of Scopus, the combination of the TITLE and KEY fields is employed, which allows both the titles and keywords of the articles to be covered, ensuring that the results are relevant and focused on the research topic. To further refine the search, variations in key term usage are incorporated, such as “Smart Restaurant” and “iot AND restaurant”, capturing diverse forms of topic expression. In Web of Science, the fields TI (title) and AK (keywords) were selected, ensuring a precise and specific search in the articles in the database. The incorporation of related terms, such as “Internet of Things”, “Artificial Intelligence”, and “Virtual Reality”, reflects an inclusive and detailed approach to ensure that all potential variations of the concepts related to smart restaurants are covered. The utilization of these two approaches is aimed at maximizing the coverage and relevance of the results obtained, while concurrently ensuring that the results are not redundant. This adaptation to the distinct characteristics inherent to each platform is a crucial aspect of the methodology.

### Data management

For this bibliometric study on intelligent restaurants, the information obtained from each of the selected databases was extracted, stored, and processed using Microsoft Excel. This platform simplifies the data organization. The free VOSviewer ^®^ program was used in a similar manner ( Eck & Waltman, 2010). It allows the analysis and visualization of biblio-metric data and the creation of graphs representing different bibliometric indicators. In this study, the combination of Microsoft Excel ^®^ and VOSviewer ^®^ was found to be a crucial method for managing data and presenting results.

### Selection process

Based on the PRISMA 2020 guidelines ( Page et al., 2021). To determine the risk of omitting relevant studies or incorrectly classifying data, it is essential to answer the question of whether an internal automated classifier was used during the study selection and internal or external validation processes. These recommendations were followed in the present study, which used Microsoft Excel ^®^ automation tools as the internal mechanism for the selection process. All study investigators contributed to the development and improvement of this tool, which they then independently used to apply the inclusion and exclusion criteria. Through the convergence of the results of the application of the tool, this collaborative approach was adopted with the aim of minimizing the risk of omitting relevant studies or incorrect classifications, ensuring integrity and accuracy in the selection of studies for current restaurant bibliometrics. intelligent.

### Data collection process

Following the established guidelines, they described the procedures for obtaining or verifying data directly with the original investigators of the studies, any procedures for doing so, and, if applicable, details about the automation tools used in data collection ( Page et al., 2021). This includes the number of reviewers involved in the process, their degree of independence, and the procedures for doing so. These guidelines were strictly followed in the context of smart restaurant studies. To ensure efficiency and consistency in the extraction of information, Microsoft Excel ^®^ was used as an automated tool for data collection of the reports obtained from the two selected databases. In addition, all authors took on the role of reviewers and completed this task independently. Subsequently, a collective data validation process was implemented in which the authors worked together to validate and authenticate the results, ensuring a high level of consistency and reliability in the collection of bibliometric data.

### Data elements

An exhaustive search was conducted to find all relevant results and articles related to the research objective in the context of the current bibliometric research on smart restaurants. This was done using specific search equations created for each database, covering all articles that mentioned the topic of interest and ensuring that all relevant research was included. It is important to note that if any of the identified articles contain ambiguous or missing information, "non-relevant texts" are excluded from consideration. This was done to ensure that only findings that add to the understanding of the smart restaurant knowledge base and meet predefined relevance criteria are included to maintain consistency with the purpose and scope of the research.

### Study risk of bias assessment

All the authors worked diligently and collaboratively to collect data and assess the risk of bias in the included studies as part of the current bibliometric review of smart restaurants. Microsoft Excel ^®^, an automated tool, was used to ensure consistency and uniformity in the process. This method ensured the quality and integrity of the results obtained and contributed to the reliability of the ongoing bibliometric research by allowing a rigorous and standardized assessment of possible biases in the included studies.

### Effect measures

It is imperative to acknowledge that effect measures characteristic of primary research, such as risk ratios and mean differences, are ill-suited for this bibliometric research on smart restaurants. Consequently, the results were analyzed and synthesized using bibliometric tools, such as the number of publications, the volume of citations, and the frequency of keyword use. These tools are more relevant for analyzing trends in the scientific literature. The data management and analysis were performed using Microsoft Excel ^®^, a software that ensured the consistency and accuracy of the process. Additionally, although not translated into measurements, VOSviewer ^®^ was used to explore thematic associations, identifying nodes and networks that provided a deep understanding of the dynamics and evolution of research in this field.

### Synthesis methods

A procedure was used in this bibliometric research on smart restaurants to select studies that could be included in the synthesis. This involved collecting information on the attributes of a document, such as the number of publications, citations, and keywords, and comparing them with the predetermined inclusion criteria. In addition, data preparation techniques were used, including imputing missing summary statistics and performing data conversion where necessary. Bibliometric indicators of quantity, quality, and structure were used in the presentation and synthesis of the results according to predetermined guidelines. These indicators were automatically applied to all documents that survived the three exclusion stages ( Durieux & Gevenois, 2010). Using this methodology, studies included in the bibliometric review were selected and analyzed for consistency and objectivity.

### Assessment of reporting bias

In the context of this bibliometric research on smart restaurants, it is important to be aware of possible information biases resulting from the use of synonyms found in thesauri, such as IEEE. These biases may be related to the lack of results in the synthesis. This vulnerability is reflected in the inclusion criteria, search approach, and data collection, where the choice of certain terms may affect the exclusion of relevant documents that use different vocabularies. Therefore, while bibliometrics must be accurate, the exclusion of irrelevant texts may result in the loss of important data for building comprehensive knowledge on the topic. This should be considered when interpreting the results and evaluating possible biases in bibliometric analyses.

### Assessment of certainty

**Figure 1.**
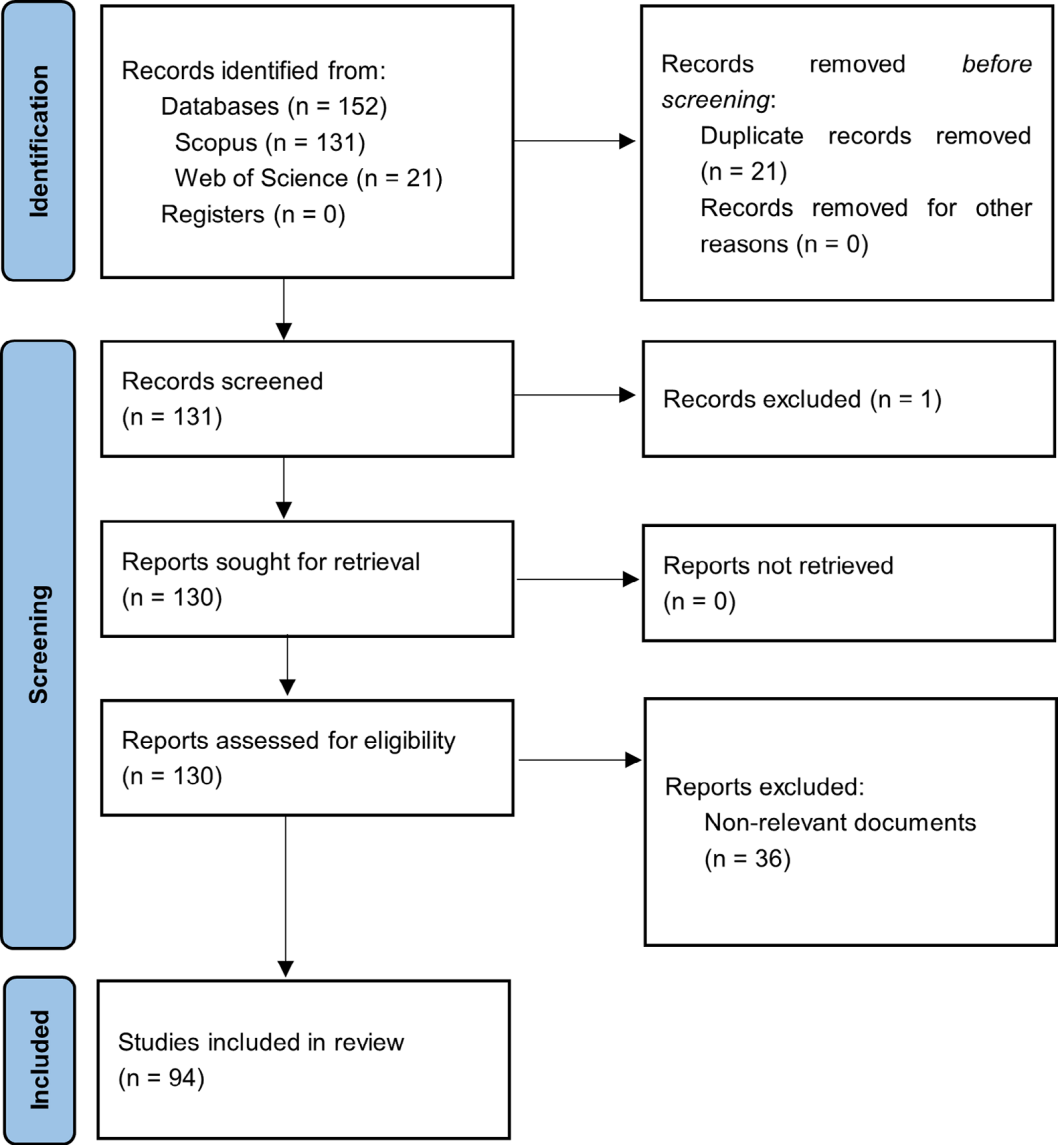
PRISMA flow chart. Own elaboration based on Scopus and Web of Science.

This bibliometric study on Smart Restaurants includes a comprehensive assessment of the certainty of the body of evidence. Here, certainty is assessed globally as opposed to primary studies, where certainty is assessed individually. This was achieved by applying independent inclusion and exclusion criteria, defining and analyzing bibliometric indicators, and identifying and disclosing potential biases resulting from the design of the methodology. In addition, the limitations of the study are discussed in the Discussion section, providing a com-prehensive assessment of the accuracy and reliability of the bibliometric results. This im-proves our understanding of the validity and substance of the conclusions. Figure 1 summarizes the methodological design.

In the first phase of this bibliometric study on Smart Restaurants, articles were identified based on the search strategy used for each selected information source, and duplicate records were eliminated. The three exclusion phases listed in the methodology section were then carried out by applying relevance and quality standards. After a long process, 94 articles that met the established criteria were identified and selected to form the main corpus of this bibliometric study.

## Results

**Figure 2.**
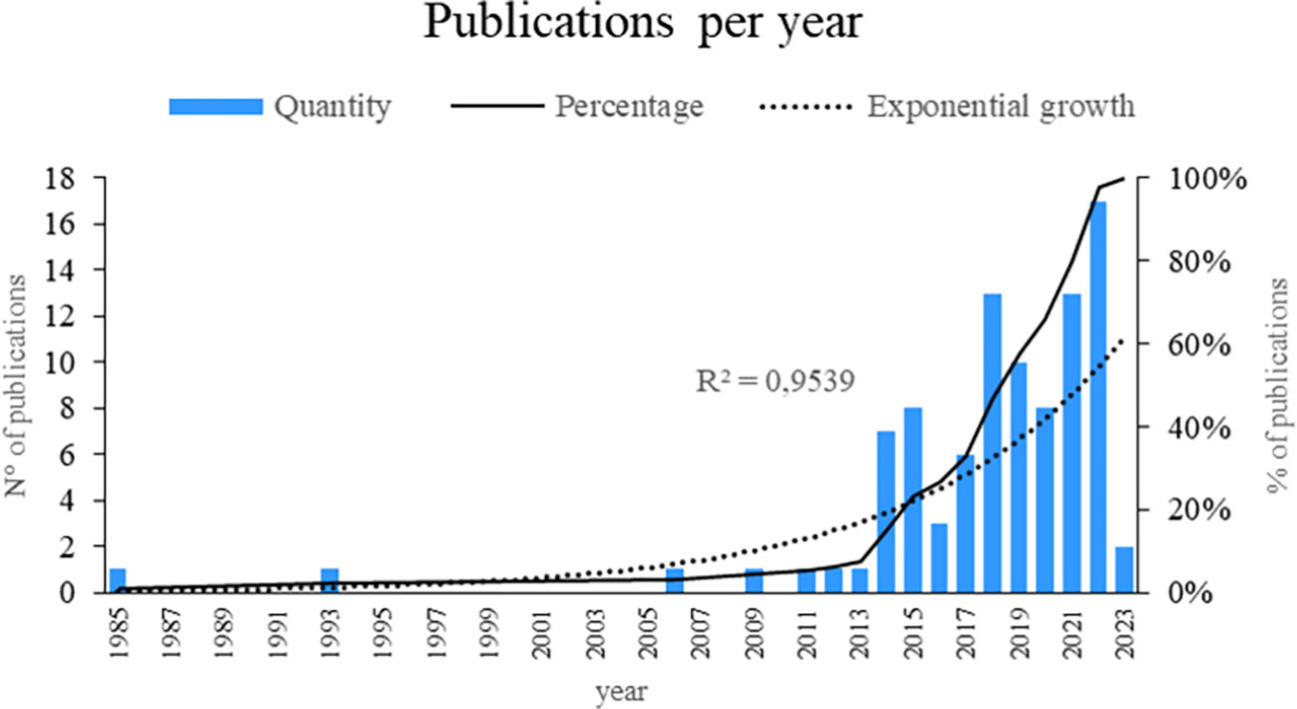
Publications by year. Own elaboration based on Scopus and Web of Science.

As shown in Figure 2, this bibliometric study provides a fascinating window for the evolution of smart restaurant research. The data showed a remarkable increase of 95.39 percent, suggesting an exponential growth pattern in the publication of articles related to this topic. Notably, the years with the highest number of publications are 2022, 2021, and 2018, suggesting that interest and research activities around smart restaurants increased significantly during these particular years. This may indicate the current relevance and timeliness of the topic in the scientific community. This temporal analysis sheds light on the changing nature of smart-restaurant research.

**Figure 3.**
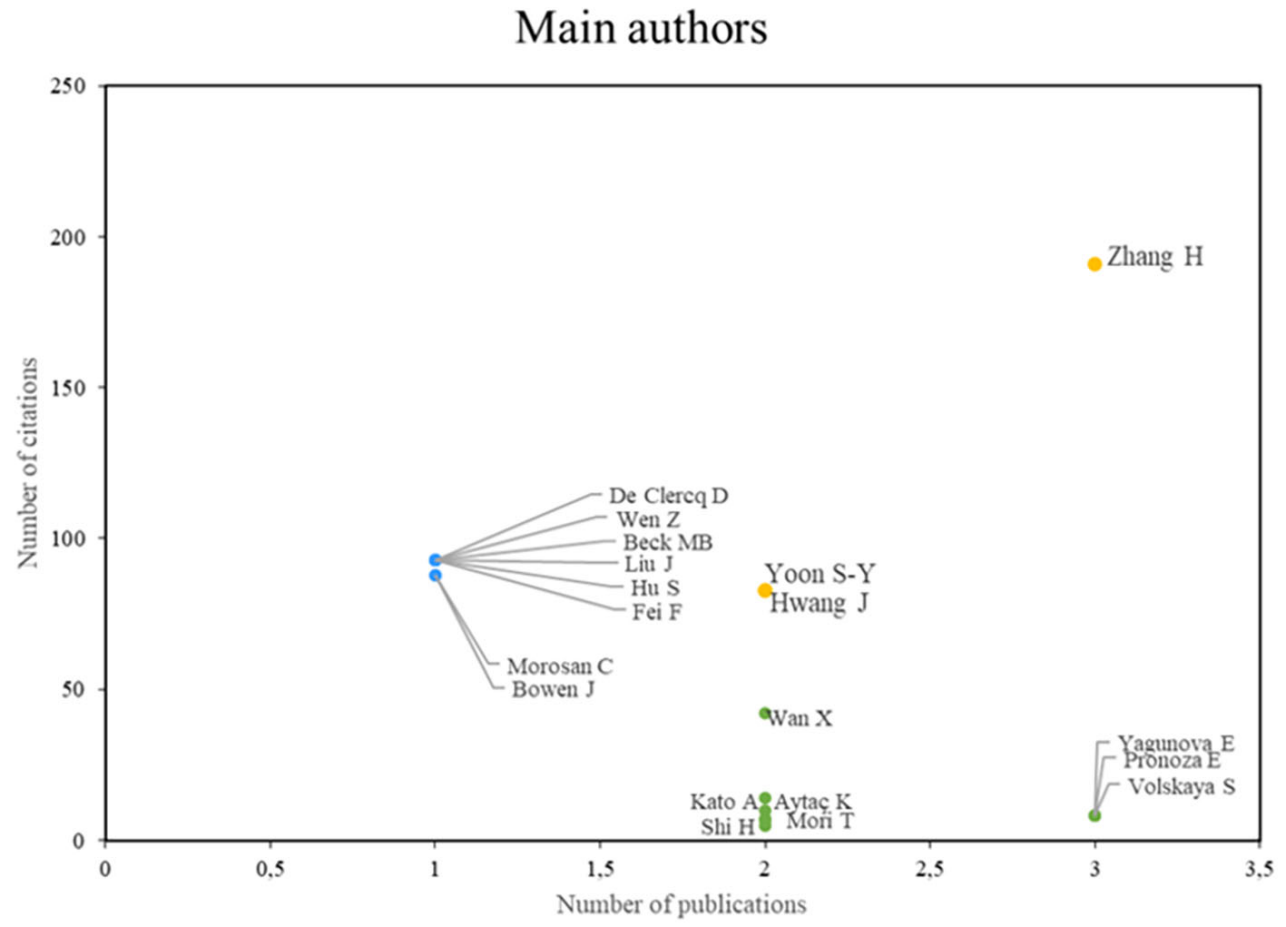
Main authors. Own elaboration based on Scopus and Web of Science.

According to the analysis of the main authors of this bibliometric study on smart restaurants, three groups can be identified. First, we can identify authors, such as Zhang, Yoon, and Hwang, who stand out in terms of their scientific productivity and the importance of their research, establishing them as leading figures in the field, as shown in Figure 3. Conversely, some authors (e.g., De Clercq, Wen, and Beck) value the impact and caliber of their work, even if their productivity rate is low. The third group of authors, Wan, Kato, and Aytac, for example, is characterized by high scholarly productivity, although not always in terms of citations, which enables them to actively advance our understanding of smart restaurants. This authorship analysis provides a deeper understanding of the current state of research in this field.

**Figure 4.**
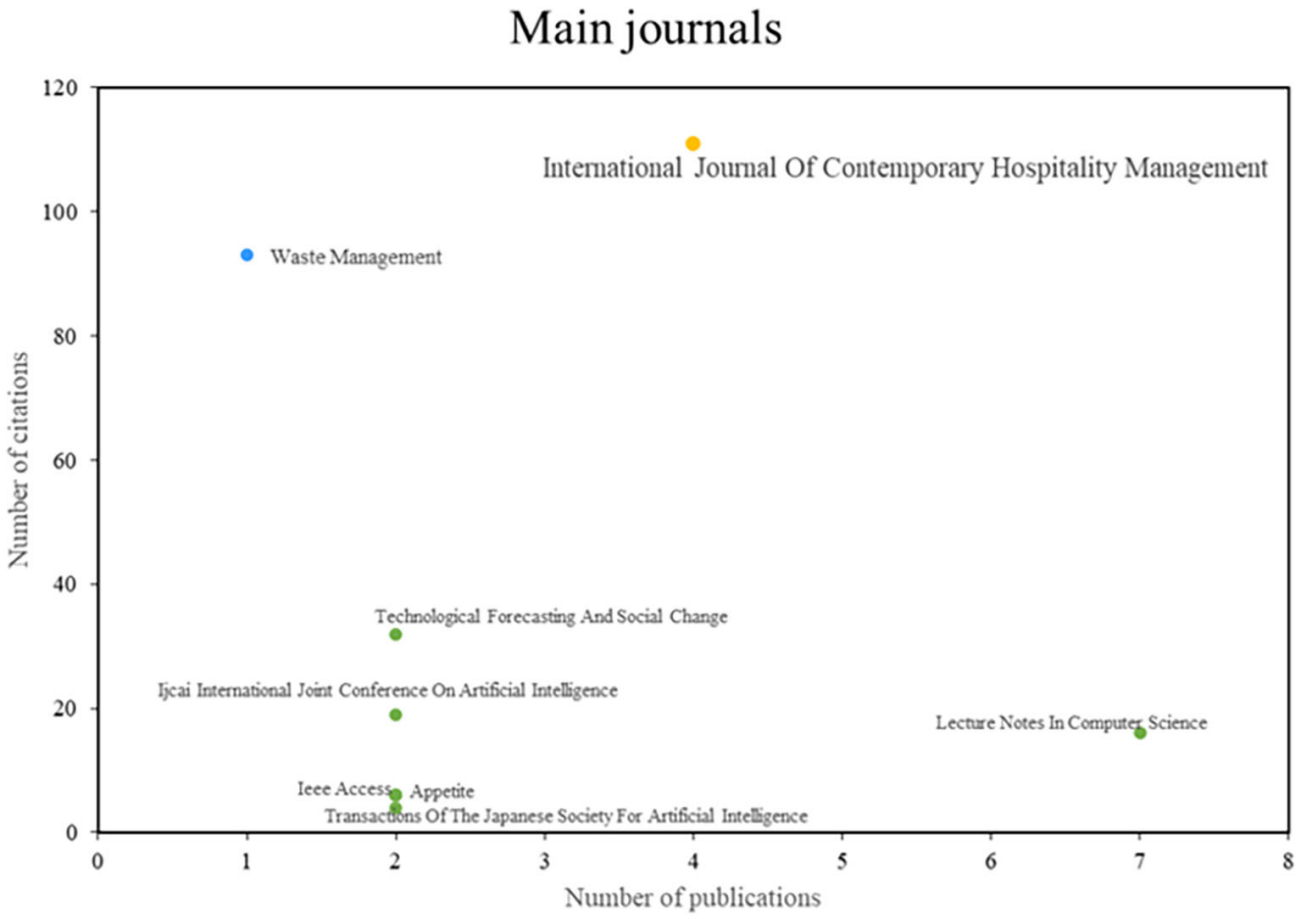
Major journals. Own elaboration based on Scopus and Web of Science.

After analyzing the main journals of this bibliometric on smart restaurants, they were able to observe three different groups (See Figure 4). First, publications such as the International Journal of Contemporary Hospitality Management stand out for their impact and productivity in the scientific community and have established themselves as leaders in the industry. On the other hand, there are publications on Waste Management, which are recognized for their impact and publication quality despite having a lower productivity index. Finally, the third group of journals is characterized by high scientific productivity, but the amount of research published rather than the number of citations is a better indicator of a journal’s influence. Examples of these journals are Technological Forecasting and Social Change and the Ijeai International Joint Conference on Artificial Intelligence. This journal analysis provides a deep understanding of the editorial dynamics in this research area.

**Figure 5.**
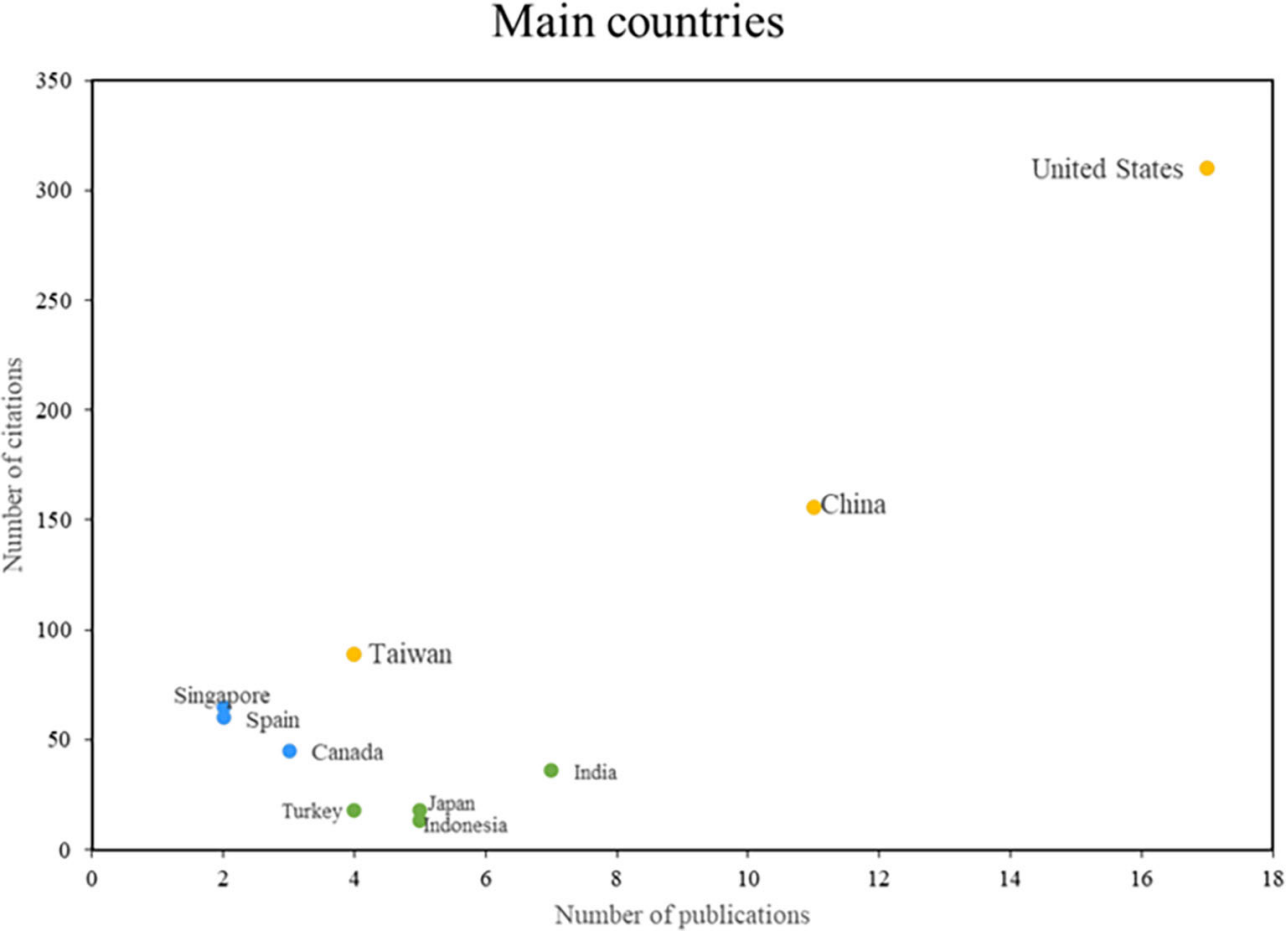
Main countries. Author's elaboration based on Scopus and Web of Science.

In the analysis of the main countries in this bibliometric study of smart restaurants, three groups can be distinguished as can be shown in Figure 5. First, some nations stand out in terms of productivity and scientific impact, such as the United States, Taiwan, and China, consolidating their positions as leaders in this field of research. On the other hand, there are countries such as Singapore, Spain, and Canada, which despite having a lower productivity index, are recognized for their importance and caliber. The impact of the third group of nations, including India, Japan, and Indonesia, is measured more by the volume of research published than by the number of citations. This places these countries in a position to actively contribute to the body of knowledge on smart restaurants. The country analysis provides a comprehensive view of the geographic distribution of research in this area.

**Figure 6.**
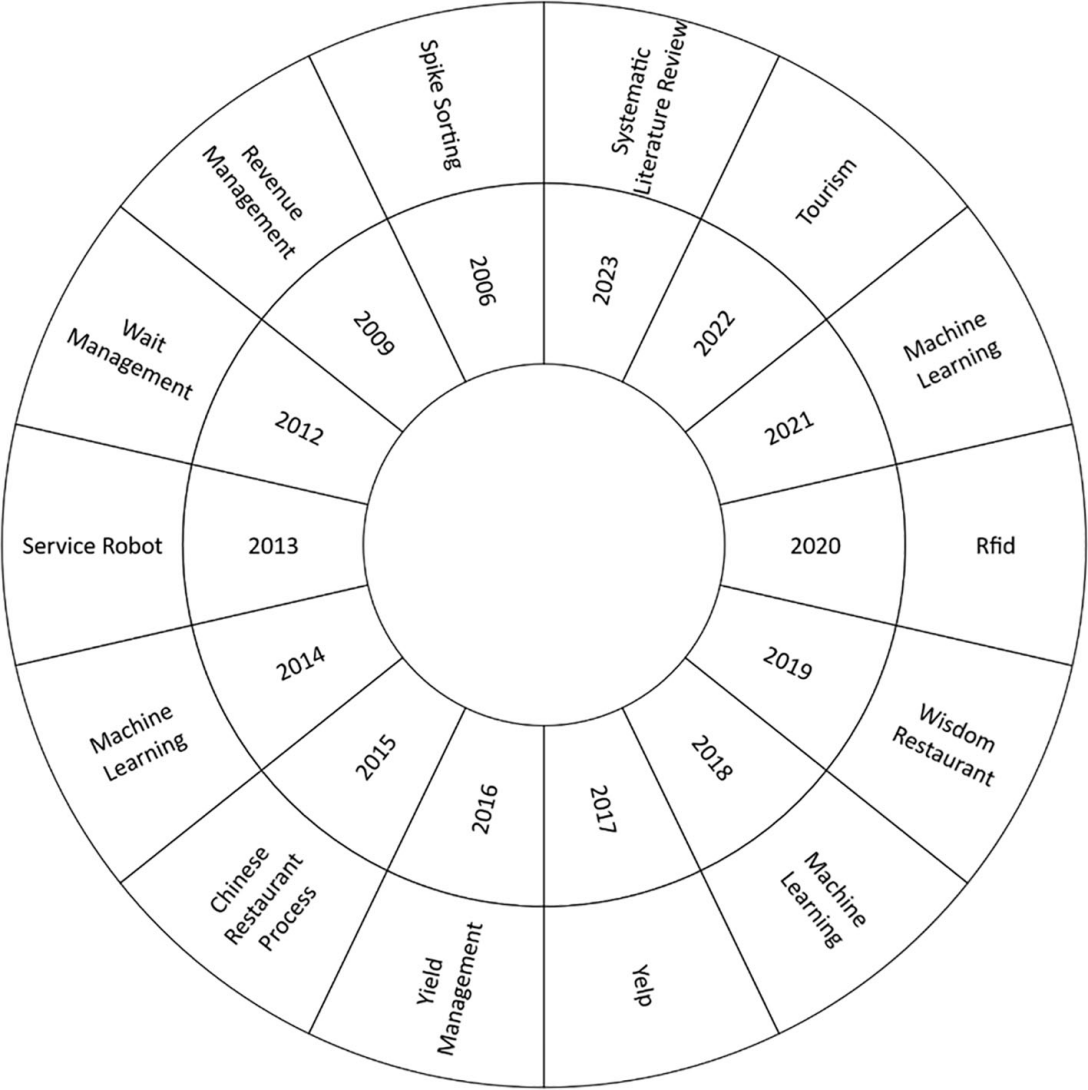
Thematic development. Own elaboration based on Scopus and Web of Science.

The current study, as shown in Figure 6, focused on the most used keyword in each research year to perform a comprehensive analysis of the thematic evolution in the Smart Restaurant literature between 2006 and 2023. As a starting point, it should be noted that 2006 is notable for the emergence of ideas such as “spike sorting”. Emerging topics such as “Radio-Frequency Identification” (RFID)”, “Machine Learning”, “Tourism”, and “Systematic Literature Review” become more prevalent over the years, reflecting changing research dynamics and areas of interest. This thematic analysis provides valuable information on the current developments and trends in the field.

**Figure 7.**
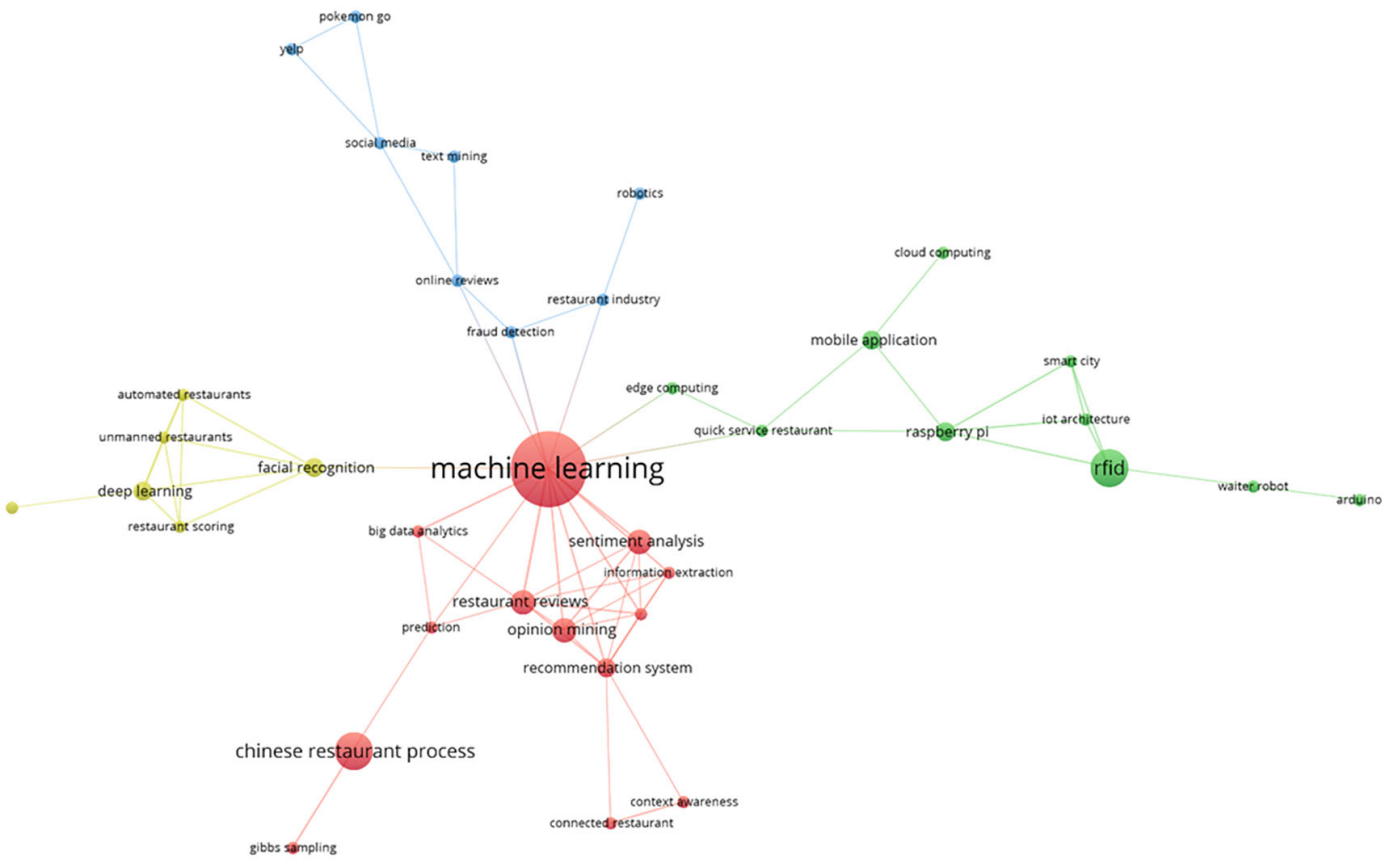
Keyword co-occurrence network. Own work based on Scopus and Web of Science.

The main keyword co-occurrence network is represented by these bibliometrics in four thematic groups, as shown in Figure 7. The red group, which includes terms such as “machine learning”, “big data analysis”, “opinion mining”, “Chinese restaurant process”, “prediction”, and “information extraction”, is the most prominent. The green group includes terms such as RFID, smart city, mobile application, edge computing, waiter robot, Arduino, and quick-service restaurant. A visual representation of the thematic interconnectedness in the scientific literature on this topic is provided by the identification of additional clusters in the area of smart restaurants, which are highlighted in blue and yellow, respectively. These groups capture the different aspects of conceptual affinity.

**Figure 8.**
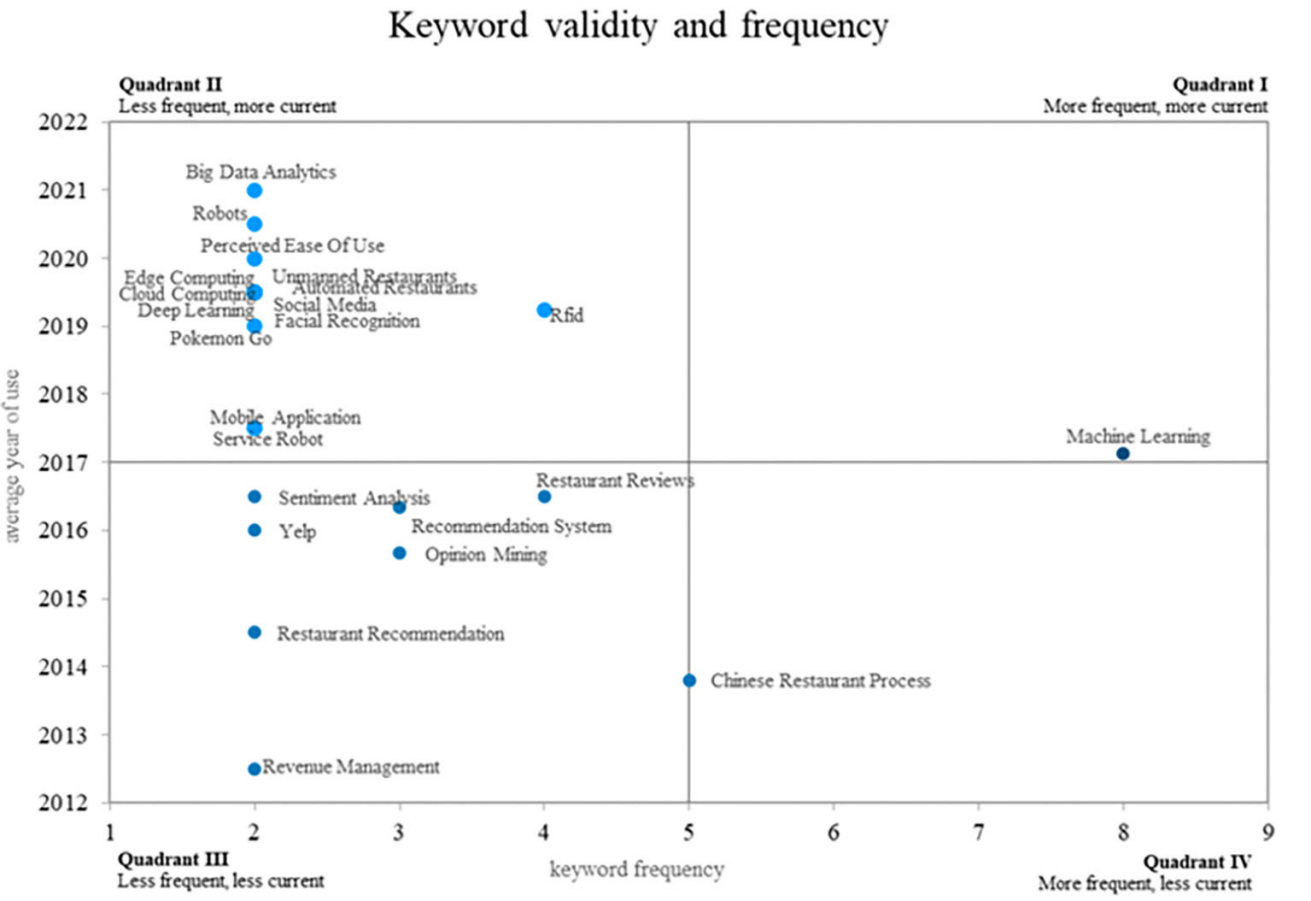
Validity and frequency of keywords. Own elaboration based on Scopus and Web of Science.

This bibliometric study of smart restaurants uses an innovative method to create four different quadrants using a Cartesian plane that combines the frequency of keyword use on the x-axis and the validity of that use on the y-axis. This method is illustrated in Figure 8. Quadrant 4 contains ideas that show a decreasing trend in use over time, as shown by terms such as “Chinese restaurant process. Quadrant 2 contains words that, although they appear infrequently, have great relevance in the recent literature, placing them in the category of emerging words. “Big data analytics, robots, edge computing, and cloud computing are some examples of these keywords. Last but not least, Quadrant 1 includes well-established and expanding ideas such as “machine learning”, which is still widely used and has applications in smart restaurant research. This method provides a distinctive visual representation of keyword dynamics in relevant scientific literature.

## Discussion

The Discussion section plays an essential role in the bibliometric analysis of smart restaurants. First, it thoroughly examines the research results, breaking down trends, patterns, and key findings. On the other hand, it provides a platform to explore the practical implications of these findings by identifying possible applications in the smart restaurant industry. Furthermore, it highlights the methodological limitations of the study, presents a classification of keywords according to their function in the literature, and highlights the main gaps in the research. Finally, it establishes a research agenda that identifies priority areas for future studies in the field of smart restaurants, thereby contributing to the understanding and continuous development of this topic.

### Analysis of the growth of the scientific literature on smart restaurants

A meticulous examination of the scientific literature pertaining to smart restaurants from 2018 to 2022 reveals not only substantial advancements but also persistent challenges in the integration of emerging technologies into this sector. In 2022, for instance, a substantial surge in research was observed on the impact of digital technology on the food environment, signifying a heightened cognizance of technological innovations’ repercussions on the restaurant industry ( Granheim et al., 2022). This finding is crucial, as it implies that the transition towards smart restaurant models is far from superficial; rather, it is linked to a profound re-evaluation of how technology can transform customer experience and restaurant management. Nevertheless, the relationship between service quality and smart technologies, as identified in the study by Wong et al. (2022), underscores the necessity to strike a balance between technology adoption, customer satisfaction, and expectations. That is, although technology can improve efficiency and personalization, its impact on the perception of service quality remains a critical area of research.

In 2021, the integration of augmented reality and artificial intelligence in the catering sector, as evidenced by the studies conducted by Batat (2021) and Blöcher and Alt (2021), signaled a growing trend towards the incorporation of advanced technologies. However, these advancements also underscore the intricacies inherent in their integration. Despite their potential to enhance the customer experience, the implementation of technologies such as augmented reality and robotics can generate resistance due to the perception of technological invasion in traditional interactions with service providers. This discrepancy underscores the necessity for a multifaceted approach to integration, necessitating not only technological advancements but also a transformation in organizational culture and a shift in consumers’ acceptance of an increasingly automated experience.

The 2018 analysis centered on the management of food waste through the IoT and warnings concerning automation ( Wen et al., 2018; Bowen & Morosan, 2018). This analysis anticipated that a technological transformation was underway in the sector, but that it still faced barriers related to sustainability and social acceptance. While technology has the potential to enhance the efficiency and sustainability of restaurants, it also gives rise to questions concerning the future of labor in the industry and the ethical implications of increased automation. This analysis is particularly pertinent in the present context, as smart restaurants are progressively integrating technological solutions for resource management and enhancing customer experience.

A final point of note is that the trend analysis demonstrates exponential growth in research on smart restaurants, similar to that seen in other service-sector industries. However, the fact that the adoption of emerging technologies in smart restaurants is still relatively low compared to sectors such as tourism and hospitality reveals a disconnect between the availability of technological solutions and their effective implementation in the sector. This observation prompts critical inquiries into the scalability of technological solutions within the restaurant sector. The findings suggest that while the technology is prepared, its adoption and the transition to digital business models may be hindered by factors such as financial constraints, staff training, and resistance to change among restaurant owners and customers.

### Analysis of research references on smart restaurants

The contributions of Zhang, Yoon, and Hwang have been instrumental in propelling the development of the smart restaurant domain, with a particular focus on food waste management and the enhancement of customer experience. Zhang’s research on food waste management using the IoT in restaurants, as outlined in the paper “Design, Implementation, and Evaluation of an IoT Network System for Food Waste Management in Restaurants” ( Wen et al., 2018), has been highlighted as an example of technological innovation in the restaurant industry. This research addresses a critical sustainability concern in the restaurant sector, demonstrating that IoT implementation can lead to substantial reductions in food waste and improvements in operational efficiency. This is of paramount importance for ensuring environmental and economic sustainability. However, the impact of this technology may be contingent upon the technological infrastructure available in restaurants, which could impede its adoption in resource-constrained establishments.

Concurrently, Yoon and Hwang’s research has been acknowledged for its exploration of the interplay between privacy concerns, spatial configuration, and customer experience in restaurant settings. Noteworthy studies include “Desired Privacy and the Impact of Crowding on Customer Emotions and Attraction-Avoidance Responses: Waiting in a Virtual Reality Restaurant” ( Hwang et al., 2012) and “Where Would You Like to Sit? Understanding Customers’ Privacy-Seeking Tendencies and Location Behaviors to Create Effective Restaurant Environments” ( Hwang & Yoon, 2009) offer valuable insights into how spatial design can influence customer emotions and behaviors. This line of research is pivotal in enhancing customer experience, as it enables a more customized approach to designing environments that diners find comfortable. However, a salient challenge in the execution of such studies is their foundation on controlled or virtual scenarios, which may not fully capture the intricacies inherent in real-life restaurant environments.

De Clercq, Wen, and Beck have also had a significant impact through their collaboration in the aforementioned study on the Internet of Things (IoT) for food waste management ( Wen et al., 2018). Their involvement underscores the significance of a multidisciplinary approach in addressing complex problems, such as waste management in the restaurant industry. However, despite the innovation in their proposal, the widespread adoption of IoT across all restaurants could be hampered by barriers such as implementation cost and staff training, which limit its applicability in such a diverse sector.

Noteworthy publications in esteemed journals such as The Journal of Contemporary Hospitality Management and Waste Management have played a pivotal role in disseminating these advancements. Notably, the article by Hwang et al. (2012) offers a thought-provoking perspective on the management of emotions in the context of virtual reality restaurants, shedding light on the intricate relationship between privacy, spatial dimensions, and customer emotional responses. Additionally, the integration of artificial intelligence in data management, as evidenced by the study by Lee et al. (2021), signifies a progression towards the automation of decision-making, a pivotal advancement in the restaurant industry. However, it is important to note the limitations of these studies, particularly concerning the generalizability of their findings and the necessity of adapting to the unique characteristics of specific restaurant contexts.

With respect to global research, findings from the United States, Taiwan, China, Singapore, Spain, and Canada offer a diverse perspective on advances in the field of smart restaurants. The United States has been a leader in the automation of processes with robots ( Bowen & Morosan, 2018), reflecting a growing trend in the industry towards robotics to improve operational efficiency. However, the implementation of this technology could raise concerns regarding the substitution of human jobs, which requires a balanced approach between automation and job preservation. In Taiwan, the emphasis on customer loyalty ( H. C. Wu & Cheng, 2018) and the integration of facial recognition technology ( Chang et al., 2019) in smart restaurants underscores the significance of personalizing the customer experience. However, the utilization of sensitive data, such as facial features, gives rise to concerns regarding privacy and ethical considerations. In China, the emphasis on service quality ( Wong et al., 2022) underscores the significance of operational excellence but also interrogates the capacity of technologies to emulate human interaction in service contexts.

Singapore has made notable strides in sentiment analysis in smart restaurants ( Truong & Lauw, 2019), which signifies an innovative approach to understanding customer opinions. However, the interpretation of this data can be intricate and contingent on the cultural and social context. Finally, research conducted in Spain ( Aguilar et al., 2018) and Canada ( Saeed et al., 2016; Lasek et al., 2016) underscores the significance of technological integration in order management and demand forecasting. This integration has the potential to enhance operational efficiency. However, it is crucial to address the adaptability of these technologies to diverse restaurant types, particularly those of a smaller scale.

### Analysis of the thematic development of smart restaurants

The concept of “spike sorting”, which was mentioned in the early years of smart restaurant research, focused on solving the challenges of data management and information categorization in restaurant systems ( Wood et al., 2006). This approach, while foundational at the time, highlighted the technological limitations of the era, as it concentrated on data analysis and organization without comprehensively addressing the intricacies inherent in the implementation of emerging technologies within a dynamic restaurant environment. While this methodology was pivotal in organizing critical data in real time, the evolution of technology and the integration of new tools, such as RFID and machine learning, have significantly expanded the horizons of research. The initial focus on fundamental data structures, as evidenced by the early emphasis on spike sorting, reflects the nascent stage of research in this area. However, contemporary research demands a more comprehensive understanding of the intricate interplay between customers, service, and technology.

The study of smart restaurants has undergone substantial evolution, with concepts such as RFID, machine learning, tourism, and systematic literature reviews acquiring considerable relevance. The advent of Radio Frequency Identification (RFID) technology has profoundly impacted inventory and logistics management in the restaurant industry, facilitating real-time tracking of products and enhancing operational efficiency ( Wong et al., 2022). However, the effective implementation of RFID technology in the restaurant sector is hindered by challenges related to the existing technological infrastructure, particularly in restaurants with limited resources. Moreover, the reliance on RFID for inventory management could diminish the flexibility of traditional systems, which still rely on manual methods for stock control. Consequently, while the potential of RFID is indisputable, its success on a large scale is contingent upon the successful navigation of challenges related to costs and training.

Concurrently, the integration of machine learning algorithms has enabled the personalization of the customer experience, encompassing menu recommendations, reservation management, and other services. This development has not only elevated customer satisfaction levels but also enhanced operational efficiency within restaurants ( Wong et al., 2022). This development is noteworthy; however, it gives rise to inquiries concerning the boundaries of automated personalization and its potential implications for human interaction, a fundamental component of the hospitality industry. While machine learning enhances the precision of recommendations and optimizes processes, it has the potential to create a disconnection between customers and staff, which would be counterproductive in a sector that has historically relied on the human touch to deliver a distinctive service experience. The integration of these technologies necessitates a careful balancing act between automation and human interaction, a challenge that remains to be fully resolved.

The realm of tourism and hospitality has emerged as a pivotal domain for research into smart restaurants, as technological advancements are profoundly altering the manner in which tourists interact with restaurant services in tourist destinations. However, this approach reflects a trend towards specialization of research, which could benefit from a more comprehensive approach that connects tourists’ technological experiences with the local experience. The challenge in this context lies in adapting the technology to the cultural and social particularities of each destination, which can create obstacles in its global implementation. Consequently, research in this domain should evolve to develop customized solutions that address both local and global requirements.

Finally, the emphasis on systematic literature reviews has been crucial to understanding the current state of knowledge in the field of smart restaurants and to identifying gaps that require further research ( Singh et al., 2023). While this approach offers a comprehensive perspective on the existing research landscape, it also underscores the pressing need to transition from descriptive studies to more applied research methodologies. Such a shift would facilitate the effective integration of novel technologies into the operational and service processes of restaurants. Despite the efforts to identify gaps in the literature, the rapid pace of technological evolution means that literature reviews can quickly become outdated, necessitating a continuous approach to updating research.

### Analysis of the smart restaurant topic clusters

The results of the thematic cluster analysis reveal interesting patterns in smart restaurant research, which are grouped based on the most frequently used terms and their relationships. The initial cluster, centered on machine learning, encompasses keywords such as sentiment analysis, big data and analytics, restaurant reviews, opinion mining, recommendation systems, and prediction ( Hajek & Sahut, 2022; Lee et al., 2021). This set of terms reflects an approach focused on the application of predictive models based on data from previous consumer reviews and opinions. While this line of research possesses the potential to transform the decision-making processes within the restaurant industry by offering options that are aligned with user preferences, the analysis of these models faces several challenges, most notably the quality and representativeness of the data ( Hajek & Sahut, 2022; Lee et al., 2021). Predictive models are inherently dependent on the data they are trained on, and it is crucial to ensure that this data is diverse and addresses a broad spectrum of consumer preferences. Moreover, while personalization driven by these models promises an enhanced experience, it is imperative to acknowledge that diversity in user preferences can introduce challenges in the accuracy of predictions, particularly when dealing with customers with tastes not represented in existing databases.

Within this cluster, there are also the terms “Chinese restaurant process”, “contextual awareness”, “connected restaurant”, and “Gibbs sampling”. These terms imply an exploration of the integration of technology with the specific process of restaurant operations, particularly in the context of Chinese restaurants. The terms “contextual awareness” and “connected restaurant" indicate a focus on the dynamic interaction between real-time data and system decisions, suggesting the creation of smarter environments that adapt to the changing needs of customers. However, further exploration is necessary to assess the feasibility and scalability of these systems across diverse restaurant types. The implementation of technologies such as Gibbs sampling, which is employed in statistical inference, can be technically demanding due to the intricacy of real-time data.

The subsequent keyword cluster, which encompasses RFID, Raspberry Pi, Arduino, IoT architecture, smart cities, mobile applications, cloud computing, edge computing, and robot waiters ( Harpanahalli et al., 2020; Wen et al., 2018), underscores a mounting emphasis on the technological infrastructure that underpins smart restaurant systems. The integration of devices such as Raspberry Pi and Arduino to form an IoT architecture, along with the incorporation of technologies such as cloud and edge computing, signifies a pivotal step towards the creation of smart spaces where data is managed efficiently and accessibly. While these technologies hold great promise for enhancing the operational efficiency of restaurants through automation and connectivity, the challenge lies in their large-scale implementation. The integration of technologies such as robot waiters in this context gives rise to concerns regarding customer acceptance and the efficacy of robots in tasks that necessitate a high degree of human interaction. Additionally, the reliance on cloud and edge computing introduces risks related to data security and privacy, underscoring the need for measures to ensure the reliability of these systems.

Another salient cluster encompasses terms such as deep learning, facial recognition, restaurant automation, restaurant rating, and unstaffed restaurants, predominantly concerning research on restaurant automation through deep learning techniques based on restaurant rating data ( Bonela et al., 2023; Dampage et al., 2021). This cluster underscores a focus on enhancing operational efficiency and personalization through the integration of cutting-edge technologies. While the potential of deep learning in automation is substantial, ethical and practical considerations must be taken into account, such as the potential impact on privacy with facial recognition technology. Additionally, the complete automation of restaurants gives rise to significant concerns regarding the social and economic ramifications of replacing human labor, a matter of particular pertinence within the restaurant industry.

The final cluster encompasses terms such as “fraud detection”, “restaurant industry”, “robotics”, “online reviews”, “text mining”, “social media”, “Yelp”, and “Pokémon Go”. This set of words suggests that research in this area also covers aspects related to safety and reputation in the restaurant industry, especially through text mining on platforms such as Yelp and social media. This allows for the identification of patterns and the detection of fraud. The inclusion of “Pokémon Go” in this cluster underscores the integration of gamification elements and the development of innovative customer experiences within the restaurant environment. However, it also prompts the inquiry into the extent to which such technologies are viable and genuinely advantageous for the industry in the long term. While gamification can serve as a compelling strategy to attract customers, its effectiveness is contingent upon its proper implementation and the extent to which users are willing to adopt such experiences.

### Analysis of frequency and conceptual validity around smart restaurants

The concept of the “Chinese Restaurant Process” (CRP) was located in quadrant 4 of the Cartesian plane, indicating a decrease in its frequency of use compared to previous periods in the field of smart restaurant research. The CRP is a statistical technique employed in data modeling, particularly within the domains of artificial intelligence and machine learning. Historically, CRP has been applied in contexts such as neural signal processing and decision making in dynamic environments ( Wood et al., 2006). It has also been used to improve reinforcement learning algorithms ( Shi et al., 2018), where it is used in the context of reinforcement learning to improve decision-making efficiency.

The CRP is a statistical technique that, although originally developed to model group assignment in stochastic processes, has broader applications in areas such as machine learning and decision making in dynamic environments. In this context, the aforementioned article addresses an approach related to reinforcement learning, specifically the improvement of Dyna-Q algorithms. These algorithms benefit from the CRP structure by applying its principles for decision optimization based on previous experiences.

While the direct relationship between CRP and smart restaurants is not explicit, the CRP methodology has been incorporated into machine learning models across various domains, including those involving dynamic interactions with intelligent systems, such as those prevalent in the restaurant industry.

However, as smart restaurant research has advanced, CRP may be becoming less relevant in this specific context. This decline may be indicative of shifting research trends and a shift in focus towards more pertinent concepts within the smart restaurant industry, such as RFID and machine learning. This suggests that smart restaurant researchers are exploring new directions and adapting their approaches to meet technological and industrial needs.

The shift in focus is evidenced by the increased prevalence of novel concepts in the domain of smart restaurants, as evidenced by the prominence of terms such as big-data analytics, robots, edge computing, and cloud computing in both the present and the near future. These terms indicate technological trends and the need to adapt to ever-changing business environments.

### Classification of keywords related to smart restaurants according to their functions

Table 1 presents a classification of emerging and growing keywords related to the development of smart restaurants, according to their functions. This allows the identification of the main characteristics and applications associated with each category, highlighting constantly growing areas of interest and prominent approaches in current research.

**Table 1.** Classification of keywords according to their function. Own elaboration based on Scopus and Web of Science.

Keyword	Associated Tools	Applications	Characteristics
Machine Learning	Big Data Analytics	Restaurant Review	Leverage machine learning from user review data on the web and social networks for predictive modeling and decision making
		Restaurant reviews	
		Information Extraction	
		Sentiment Analysis	
		Text mining	
Restaurant automation	Deep Learning	Facial Recognition	Using deep learning techniques to program robotic waiters to assist diners, automate processes, and improve service times.
		Waiter robots	
		Fast service in restaurant	
IoT Architecture	Raspberry PI	Restaurant Industry	Use programmable devices and RFID technology to implement an IoT architecture to launch the smart restaurant, based on the provision of data in the cloud and the generation of predictive models.
	RFID		
	Arduino		
	Cloud Computing		

### Theoretical implications

Conducting bibliometrics on smart restaurants with a focus on different dimensions, such as the frequency of publications over the years, identification of prominent theoretical references, monitoring of thematic evolution, analysis of the co-occurrence of keywords, exploration of emerging and growing keywords, and identification of unexplored areas of research, has important theoretical implications for the advancement and understanding of this field of study.

First, analyzing the frequency of publications over the years provides an overview of the dynamics of research over time. This allowed us to identify periods of greater research activity, thus revealing the relevance and interest in the topic at different times. The identification of key years, such as 2022, 2021, and 2018, with a high number of publications suggests the importance of these periods for the development of smart restaurant research.

Identification of the main theoretical references in bibliometrics provides a deeper understanding of the theoretical and methodological approaches that have guided research in this field. Authors such as Zhang, Yoon, Hwang, De Clercq, Wen, and Beck stand out in terms of productivity and impact, indicating their significant contribution to the existing body of knowledge.

Furthermore, the analysis of thematic evolution in bibliometrics reflects how smart restaurant research evolved from initial concepts such as “spike sorting” in the early years to emerging and consolidated areas such as “machine learning”, RFID, “edge computing”, and “big data analytics”. This analysis not only helps to understand the historical trajectory of research but also identifies current and future trends in the field.

From a theoretical standpoint, this bibliometric analysis contributes to our understanding of the evolution of the field by identifying the main trends, thematic clusters, and emerging concepts. Furthermore, it underscores the necessity for theoretical frameworks that elucidate the interplay between technological adoption and consumer behavior in the context of smart restaurants.

Keyword co-occurrence analysis reveals the conceptual structure of the research field and how key terms are related. The identification of thematic clusters and highlighted keywords within each cluster provided an overview of the most relevant areas of focus in smart restaurants.

The analysis of emerging and growing keywords allows for the identification of concepts that are gaining importance and are expected to have a significant impact on future research. Terms such as “robots”, “edge computing”, and “cloud computing” emerge as areas of emerging interest suggesting new directions for research.

Finally, the identification of unexplored research areas is a crucial implication of bibliometrics. By analyzing the gaps in the existing literature, valuable guidance is provided to researchers and academics interested in addressing underexplored areas or identifying unresolved research questions in the field of smart restaurants. These gaps can serve as a starting point for future research and contribute to the theoretical and practical advancement of the discipline.

### Practical implications

Conducting bibliometrics on smart restaurants with a focus on thematic evolution and keyword analysis has practical implications of great relevance for both the restaurant industry and academic community. The transition from research focused on the concept of spike sorting to emerging areas, such as RFID, machine learning, tourism, and systematic literature review, reflects a growing understanding of the importance of technology and data management in improving efficiency and quality in the restaurant industry. This means that industry professionals must stay abreast of emerging trends and consider implementing technologies, such as machine learning and big data analytics, to optimize decision making and improve customer experience.

The identification of thematic clusters and the conceptual relationship between key terms, such as machine learning, big data analytics, and restaurant reviews, highlight the importance of interdisciplinary research and collaboration between researchers and professionals from different fields. This suggests that smart restaurants can benefit from a comprehensive approach that integrates advanced technologies with a deep understanding of customer preferences and opinions, which in turn can influence strategic decision-making and service quality management.

Analyzing keyword frequency and relevance provides valuable insights into current trends in the industry. The emergence of concepts such as big data analytics, robots, edge computing, and cloud computing underscores the importance of digitization and automation in the restaurant industry. This suggests that smart restaurants can improve their operational efficiency and service quality by implementing advanced technologies such as robots and cloud-based management.

However, the continuous increase in the frequency of keywords related to Machine Learning indicates that this approach remains relevant and crucial in the research and application of technologies in smart restaurants. The practical implications of this finding are significant as they highlight the importance of training and adopting machine learning models for a variety of applications, such as service personalization and customer data management.

In addition, bibliometrics provide insights into best practices in research and methodology in the field of smart restaurants. This knowledge can be used by researchers and academics to understand the most effective techniques and approaches for generating knowledge in this area. Likewise, the analysis of thematic development can serve as a guide to identifying areas of research that have received disproportionate attention or, on the contrary, have been insufficiently addressed, which can improve the quality and relevance of future research projects.

Another important practical implication is related to education and training in hospitality and restaurant management. The results of bibliometrics can be used to develop education and training programs to prepare students and professionals to meet the changing technological demands of the food and hospitality industry. Emerging and growing concepts, such as machine learning or big data analytics, can be incorporated into curricula to ensure that future professionals acquire the necessary skills.

The research offers practical guidance for restaurant managers and owners to effectively adopt emerging technologies in their operations. By identifying key trends and areas of focus, industry professionals can make informed decisions about implementing technological solutions that improve operational efficiency and service quality. Keyword analysis highlights the importance of interdisciplinary collaboration and integrating technology with a deep understanding of customer preferences and opinions. This underscores the need for strategic management that combines technical knowledge with a customer-focused vision to enhance the dining experience and increase loyalty.

The research has a significant impact on the food and beverage industry by providing a roadmap for innovation and continuous improvement. It offers detailed insights into trends and best practices in the smart restaurant space, helping professionals keep up with technological developments and changing consumer demands. This benefits restaurant owners by improving their competitiveness and profitability, while also contributing to customer satisfaction and the sustainable growth of the industry as a whole.

The research emphasizes the significance of hotel and restaurant management training and education from a societal perspective. This is to prepare the next generation of professionals to meet the industry’s technological challenges by integrating emerging technological concepts and tools into curricula. Educational institutions can ensure that students acquire the skills necessary to thrive in an increasingly digitalized business environment. Encouraging the adoption of technologies that improve efficiency and quality of service can contribute to the creation of employment and economic development in the restaurant sector.

On a practical level, the findings suggest that restaurants that adopt technologies such as AI and data analytics can improve customer experience and optimize operational management. However, for these solutions to be effective, it is crucial that training strategies are designed for staff and the factors of resistance to change within the industry are analyzed ( Joo et al., 2023).

### Limitations

The present bibliometrics on smart restaurants, carried out using the PRISMA-2020 methodology and based on the Scopus and Web of Science databases, provides a valuable overview of research in the field. However, as is usually the case with bibliometric studies, it has certain limitations that must be considered when interpreting results. First, it is important to emphasize that the quality and completeness of the databases used may have affected the representativeness of the results. Despite efforts to cover a wide range of academic publications, some relevant research may have been absent or underrepresented in the selected databases. In addition, inherent limitations in keywords and search criteria may have influenced the selection of articles for inclusion in the analysis, which could have affected the breadth and depth of the identified research landscape.

Another limitation is related to the quality of the bibliographic data available in the databases. Errors in the cataloging of publications, lack of uniformity in the introduction of metadata, and duplication of records may have influenced the precision of the bibliometric indicators used in this study. Furthermore, differences in how publications are cited in different disciplines and contexts may have influenced keyword co-occurrence analysis and the identification of thematic trends.

Finally, it is important to recognize that while bibliometrics provide a valuable overview of the scientific production in a field, they do not have the capacity to fully capture the quality, relevance, or real impact of research. The sheer number of publications does not always reflect the depth or influence of a field of study, and it is essential to complement bibliometric findings with qualitative assessments and critical literature reviews to obtain a complete understanding of the current state of smart restaurant research.

Despite the comprehensive nature of this bibliometric analysis, it is important to note its inherent limitations. The selection of documents was based on the Scopus and Web of Science databases, which might have resulted in the exclusion of relevant studies published in other sources. Additionally, the bibliometric methodology employed precludes the execution of a meticulous qualitative analysis of the reviewed studies. To address these limitations, future research could complement the bibliometric analysis with more comprehensive systematic reviews and empirical studies that validate the findings in real-world settings.

It is imperative to underscore the distinction between this study and a conventional systematic review. A systematic review, by contrast, seeks a critical and qualitative synthesis of the literature. A bibliometric study, on the other hand, analyzes research patterns, co-occurrence networks, and trends using quantifiable data. While the set of 94 publications may appear to be limited, it aligns with the criteria established in previous bibliometric studies and offers a representative sample of current trends in smart restaurant research.

### Research gaps

Table 2 summarizes the main underexplored research areas identified in the bibliometric study of smart restaurants. These areas represent areas where future research could focus on advancing our knowledge. Highlighted areas of need include further exploration of the implementation of emerging technologies, such as artificial intelligence and the Internet of Things, in restaurant management and operations. The importance of exploring how digital experiences and customer interactions reshape the restaurant industry and how they can be optimized is also highlighted.

**Table 2.** Research gaps. Author's calculations based on Scopus and Web of Science.

Category	Investigative Gaps	Justification of the Gap	Questions for Future Researchers
Thematic Gaps	Integrating sustainability into smart restaurants	Sustainability is a key issue in the food industry, but more research is needed on how technologies in smart restaurants can contribute to economic and environmental sustainability.	How can intelligent restaurant technologies promote sustainable practices? What are the implications for economic and environmental sustainability?
	Social and Cultural Effects of Technologies in Restaurants	Despite their adoption, there is little understanding of how smart restaurant technologies affect social interactions and the perception of the dining experience.	How do technologies influence the customer experience and the authenticity of the dining experience?
Geographic Gaps	Regional context and adaptation of technologies in developing countries	Most research focuses on developed countries, leaving a gap in understanding how these technologies are applied in regional contexts and in developing countries.	How are technologies implemented in smart restaurants in regional contexts and developing countries? What are the unique challenges in these regions?
Interdisciplinary Gaps	Multidisciplinary collaboration to research smart restaurants	Because this is an interdisciplinary field, collaboration between experts in technology, sustainability, and experience design is essential, but currently limited.	How can experts from different disciplines collaborate effectively to address smart restaurant challenges? What are the advantages of a multidisciplinary perspective?
Temporal Gaps	Long-Term Evolution of Smart Restaurants	Given the rapid evolution of this field, research is needed to understand how it will evolve in the long term and which trends will prevail.	What are the expected long-term trends in smart restaurants? How will these technologies evolve in the coming years?

Notwithstanding the mounting interest in the subject, there are several lacunae in the extant literature that impede the progression of the field. A significant gap pertains to the absence of longitudinal studies that examine the long-term ramifications of the integration of technologies such as the Internet of Things, artificial intelligence, and big data on the functioning and financial viability of restaurants ( Hajek & Sahut, 2022). Moreover, the preponderance of studies identified in this bibliometric analysis focuses on developed countries, particularly China, the United States, and India, thereby creating a lacuna in the analysis of the impact of these technologies on emerging economies and small businesses.

Another critical aspect that merits attention is the fragmentation of research. While some studies address service automation through robots ( Mishra et al., 2022), others focus on user experience and technology acceptance ( Wu & Cheng, 2018). However, there is a paucity of studies that integrate both approaches to provide a comprehensive analysis of the impact of these innovations on operational efficiency and customer satisfaction.

Other areas highlighted in the table include the lack of research specifically dedicated to sustainability in smart restaurants as well as the lack of studies that take a detailed look at data management and cybersecurity in this context. The influence of cultural and regional factors on the adoption of smart technologies in restaurants is another area that is considered relevant for future research.

### Research agenda

**Figure 9.**
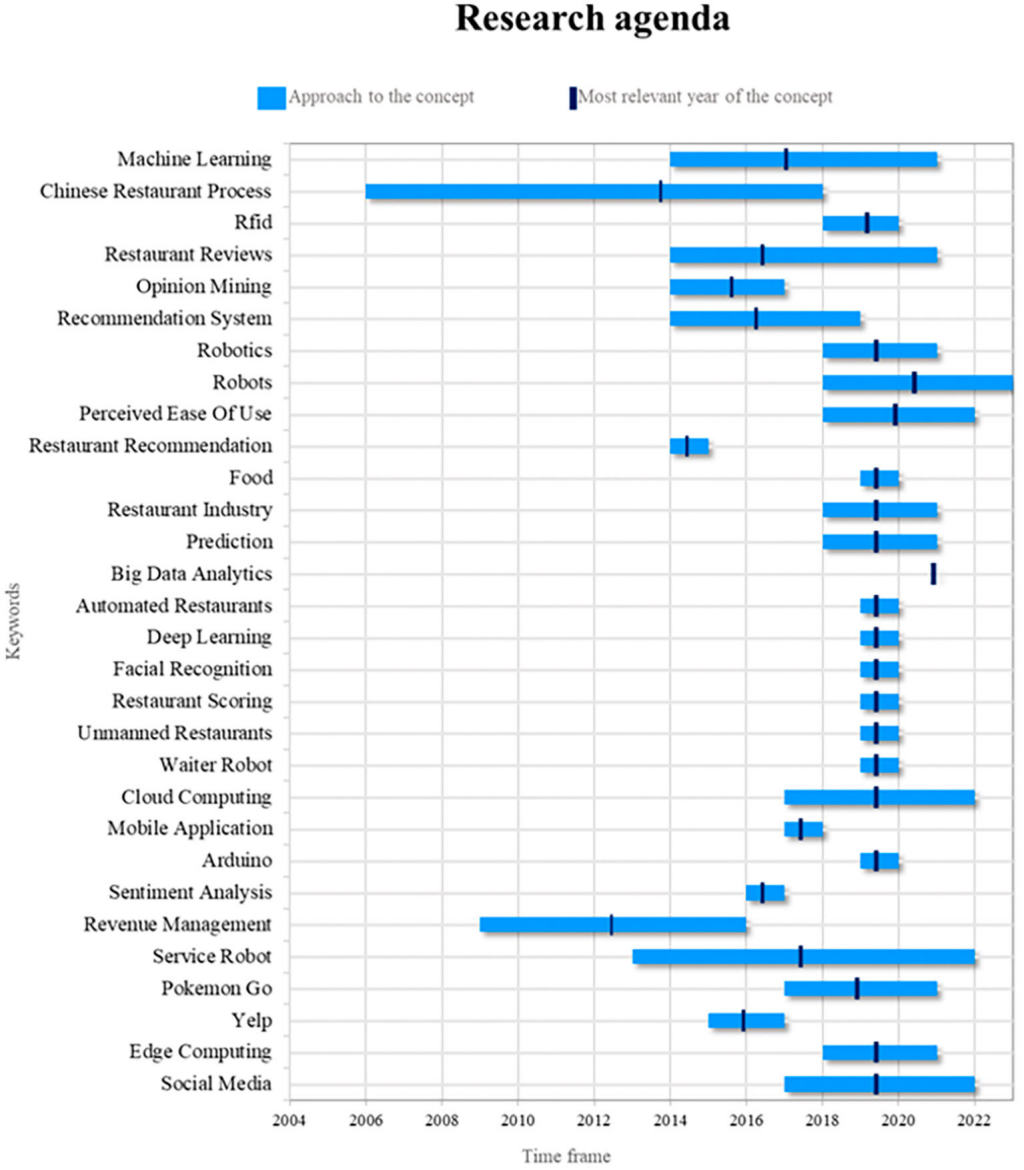
Research agenda. Author's elaboration based on Scopus and Web of Science.

Robots are essential components of the smart restaurant industry. Their relevance lies in their ability to automate repetitive tasks, improve operational efficiency, and provide customers with unique experiences. In future research, it will be possible to explore more advanced approaches to programming robots for specific tasks, such as food delivery and interaction with diners. In addition, research can be conducted on how artificial intelligence and machine learning can improve the ability of robots to adapt to changing restaurant environments, including the ability to recognize and respond in a more personalized manner to customer preferences (see Figure 9).

Another interesting aspect that could be the subject of future research is the ethics of robotics in restaurants. Addressing issues related to the privacy and security of data collected by robots as well as the impact on the quality of employment in the service industry are areas that deserve further analysis. In addition, investigating how restaurants can ensure customer comfort and acceptance of robots in their establishments is critical for promoting their adoption and ensuring their continued success.

The concept of the Chinese Restaurant Process (CRP) has been central to smart restaurant research, but its relevance has waned in recent years. However, this still offers significant potential for future research. Potential applications of CRP can be explored in resource allocation and inventory management in restaurants, with the goal of improving operational efficiency and reducing food waste. In addition, CRP can have significant implications in analyzing customer preferences and customizing menus based on order history.

On the other hand, the concept of “service robots” has become increasingly important in the context of smart restaurants. Future research could focus on how to effectively integrate service robots with CRP to improve both the customer experience and operational efficiency. In addition, restaurant review analysis is critical for decision making and customer feedback. Future studies could investigate how CRP can contribute to the automated analysis of restaurant reviews, and how to improve service quality based on customer feedback. These investigations have the potential to revitalize the relevance of CRP in the context of smart restaurants and contribute to the advancement of the industry.

## Conclusions

After analyzing the bibliometrics of smart restaurants, it is clear that interest and research in this area has experienced a significant increase in recent years. In particular, 2021 and 2022 have emerged as the most productive years in terms of scientific production on the topic. This increase is not linear; however, the number of scientific articles has grown exponentially by an impressive 95.36%. This dynamic demonstrates strong academic and professional enthusiasm for further understanding and developing this emerging industry.

In terms of the main references of the research, Zhang H, Yoon S-Y and Hwang J stand out as the most influential authors in the field, with the International Journal of Contemporary Hospitality Management serving as the main vehicle for the dissemination of these studies. It is also interesting to note the geographical concentration of scientific production, with the United States, China, and Taiwan being pioneering countries in research on smart restaurants.

At the thematic level, there has been evolution in the topics studied. In the beginning, research focused on more technical aspects such as spike sorting; today, it has diversified to cover areas such as tourism and systematic literature review. However, the main thematic clusters highlight the incorporation of artificial intelligence and data analysis techniques into the sector, with key concepts such as machine learning, sentiment analysis, and recommendation systems leading the discussion.

The field is constantly evolving, as evidenced by the emergence of keywords. Consolidated concepts, such as machine learning, coexist with growing terms, such as big data analytics, robots, perceived ease of use, and edge computing. These terms not only highlight current trends but also suggest possible directions for future research.

Finally, in the future, the smart restaurant research agenda should focus on deepening these emerging and consolidated concepts. Given the rapid evolution of this field, it is essential to maintain an up-to-date perspective and be prepared to address the challenges and opportunities that these technological and thematic changes will bring to the design and operation of restaurants.

## Ethics and consent

Ethical approval and consent were not required.

Reporting guidelines: PRISMA checklist DOI: https://doi.org/10.5281/zenodo.14146154 ( Valencia Arias and Cardona Acevedo, 2024).

Data are available under the terms of the Creative Commons Attribution 4.0 International license (CC-BY 4.0).

## Data Availability

The data availability statement for this study has been duly registered and archived in the Zenodo open data repository, which is recognized for its commitment to the accessibility and preservation of scientific data. The data and materials supported by this study are publicly available under a Creative Commons Attribution 4.0 International (CC BY 4.0) license and can be accessed at the following DOI link: https://doi.org/10.5281/zenodo.14146154. ( Valencia Arias and Cardona Acevedo, 2024).
