# Supplementary material for: Trends in Smart Restaurant Research: Bibliometric Review and Research Agenda
Source: F1000Res. 2025 Aug 15;13:1505. Originally published 2024 Dec 9. [Version 4] doi: 10.12688/f1000research.158066.4 (PMC12413613; doi:10.12688/f1000research.158066.4)
Supplement: Peer review history

# Reviewer report: Reviewer response for version 4

**DOI:** 10.5256/f1000research.186142.r406552

**Published:** 2025-09-05

**Authors:** Mohammad Badruddoza Talukder

**Affiliations:** 1 International University of Business Agriculture and Technology, Dhaka, Bangladesh

Thank you for the opportunity to review the paper titled *“*Trends in Smart Restaurant Research: Bibliometric Review and Research Agenda.”

 The paper addresses an interesting and timely topic; however, I would like to offer some suggestions for improvement:

1. To enhance clarity and improve the readers’ understanding, I recommend including a visual methodological framework that outlines the bibliometric process, databases used, and analytical techniques applied.
2. the introduction section lacks sufficient motivation and justification for the study. Please consider strengthening this part by highlighting the research gaps, the significance of the study, and how it contributes to existing literature.
3. It would be beneficial to add a dedicated recommendations section before the conclusion. This will allow the authors to translate their findings into actionable insights for researchers, practitioners, and policymakers in the field of smart restaurant management.
4. Please confirm that the paper complies with ethical publishing standards and does not contain any AI-related authorship or content integrity issues. I found 100% AI in abstract section.

Overall, the paper has potential, but addressing the above points will significantly improve its rigor, clarity, and impact. Wishing the authors the best of luck in refining their work.

Is the work clearly and accurately presented and does it cite the current literature?

Yes

If applicable, is the statistical analysis and its interpretation appropriate?

Partly

Are all the source data underlying the results available to ensure full reproducibility?

Yes

Is the study design appropriate and is the work technically sound?

Yes

Are the conclusions drawn adequately supported by the results?

No

Are sufficient details of methods and analysis provided to allow replication by others?

No

Reviewer Expertise:

Hotel Management, Hospitality and Tourism Management

I confirm that I have read this submission and believe that I have an appropriate level of expertise to confirm that it is of an acceptable scientific standard, however I have significant reservations, as outlined above.

# Reviewer report: Reviewer response for version 3

**DOI:** 10.5256/f1000research.183284.r390700

**Published:** 2025-07-24

**Authors:** Mingchen Li

**Affiliations:** 1 University of the Chinese Academy of Sciences, Beijing, Beijing, China

The authors have made noticeable progress since version 2. The introduction now draws on digital-economics, user-experience-design and service-management theories, providing a clearer academic positioning. The overall narrative has been re-organised around the three pillars “Intelligent Services – Customer Interaction – Operational Efficiency,” which greatly improves readability. The expanded cross-national discussion (USA, Taiwan, China, Singapore, Spain, Canada) adds welcome contextual richness.

 However, two core methodological issues flagged in my previous report remain only partially resolved: (1) the search strategy is still too narrow and internally inconsistent, and (2) no quantitative validation of the keyword-co-occurrence clusters is provided. Because these issues materially affect the reliability of the results, I maintain an “Approved with Reservations” recommendation.

 1. **Request**: expand and harmonise the terms, report the full search log (date, hits per database, final inclusion per round), and justify database exclusion (e.g. IEEE Xplore, Google Scholar), or at least provide a sensitivity analysis.

 2. **Request**: supply at minimum the modularity-Q and average silhouette values for the VOSviewer network, together with cluster size and density tables. If values are low, discuss implications for topic interpretation.

 3.Although additional theories are cited, the paper would gain from a concise conceptual framework showing how technological drivers, adoption theories (TAM/UTAUT/Service-Dominant Logic), and bibliometric findings interrelate. A schematic figure would suffice.

Is the work clearly and accurately presented and does it cite the current literature?

Yes

If applicable, is the statistical analysis and its interpretation appropriate?

Partly

Are all the source data underlying the results available to ensure full reproducibility?

Partly

Is the study design appropriate and is the work technically sound?

Yes

Are the conclusions drawn adequately supported by the results?

Yes

Are sufficient details of methods and analysis provided to allow replication by others?

Partly

Reviewer Expertise:

Artificial intelligence, energy economy

I confirm that I have read this submission and believe that I have an appropriate level of expertise to confirm that it is of an acceptable scientific standard, however I have significant reservations, as outlined above.

# Response

**Published:** 2025-08-05

**Authors:** JHOANY ALEJANDRO VALENCIA ARIAS

We thank the reviewer for his valuable contributions to the quality of the article. Below, we provide a point-by-point response:

**Comment**: 1. Request: expand and harmonise the terms, report the full search log (date, hits per database, final inclusion per round), and justify database exclusion (e.g. IEEE Xplore, Google Scholar), or at least provide a sensitivity analysis.

**Response**: The description of the search terms has been expanded and harmonized, including a detailed log with dates, results by database, and the final number of documents included per phase. In addition, the justification for the exclusion of other databases such as IEEE Xplore and Google Scholar has been incorporated into the study limitations section.

**Comment**: 2. Request: supply at minimum the modularity-Q and average silhouette values for the VOSviewer network, together with cluster size and density tables. If values are low, discuss implications for topic interpretation.

**Response**: Thanks for the suggestion. Although Q-modularity and silhouette scores aren't available in VOSviewer, we provide cluster size and link strength. These types of metrics are common in other programs like CiteSpace; however, they are not part of the research resources.

**Comment**: 3. Although additional theories are cited, the paper would gain from a concise conceptual framework showing how technological drivers, adoption theories (TAM/UTAUT/Service-Dominant Logic), and bibliometric findings interrelate. A schematic figure would suffice.

**Response**: A conceptual framework (Figure 9) has been incorporated that articulates technological drivers, adoption theories (TAM, UTAUT, and Service-Dominant Logic), and bibliometric findings.

# Reviewer report: Reviewer response for version 3

**DOI:** 10.5256/f1000research.183284.r390702

**Published:** 2025-06-24

**Authors:** Rainer Alt, Gräser Gräser

**Affiliations:** 1 Leipzig University, Leipzig, Germany; 2 Information Systems Institute, Leipzig University, Leipzig, Saxony, Germany

The points raised in the last review do not appear to have been adequately addressed. In particular, I still consider the search terms and search strategy to be problematic. Please refer to the last review.

Is the work clearly and accurately presented and does it cite the current literature?

Partly

If applicable, is the statistical analysis and its interpretation appropriate?

Not applicable

Are all the source data underlying the results available to ensure full reproducibility?

Partly

Is the study design appropriate and is the work technically sound?

Partly

Are the conclusions drawn adequately supported by the results?

Partly

Are sufficient details of methods and analysis provided to allow replication by others?

No

Reviewer Expertise:

Business information systems, Electronic Business, Digital Platforms

We confirm that we have read this submission and believe that we have an appropriate level of expertise to state that we do not consider it to be of an acceptable scientific standard, for reasons outlined above.

# Reviewer report: Reviewer response for version 2

**DOI:** 10.5256/f1000research.178017.r382076

**Published:** 2025-05-26

**Authors:** Mingchen Li

**Affiliations:** 1 University of the Chinese Academy of Sciences, Beijing, Beijing, China

1. The manuscript addresses an emerging and timely topic—smart restaurant research—through a bibliometric lens, offering practical implications for future development. However, the theoretical framework remains underdeveloped. A clearer integration of smart restaurant technologies with theories from digital economics, user experience design, and service management would significantly strengthen the academic contribution.

 2. The visualization of the co-occurrence network and thematic clusters is a strength of the paper. However, the manuscript lacks validation of the clustering quality—no modularity value, silhouette coefficient, or statistical justification is reported. Such metrics would enhance the robustness of the thematic structure.

 3. The review covers a wide range of subfields—AI, IoT, robotics, and service automation—but reads as a collection of isolated summaries. Organizing the literature around thematic pillars such as “Intelligent Services,” “Customer Interaction,” and “Operational Efficiency” would improve narrative flow and analytical depth.

 4. The manuscript highlights that most publications originate from countries such as China, India, and the United States. However, cross-national differences in regulatory environments, cultural acceptance, or technological infrastructure are not elaborated upon. Case-based comparative insights would enrich the analysis and global relevance.

Is the work clearly and accurately presented and does it cite the current literature?

Yes

If applicable, is the statistical analysis and its interpretation appropriate?

Partly

Are all the source data underlying the results available to ensure full reproducibility?

Partly

Is the study design appropriate and is the work technically sound?

Yes

Are the conclusions drawn adequately supported by the results?

Yes

Are sufficient details of methods and analysis provided to allow replication by others?

Partly

Reviewer Expertise:

Artificial intelligence, energy economy

I confirm that I have read this submission and believe that I have an appropriate level of expertise to confirm that it is of an acceptable scientific standard, however I have significant reservations, as outlined above.

# Response

**Published:** 2025-06-02

**Authors:** JHOANY ALEJANDRO VALENCIA ARIAS

We thank the reviewers and editorial team for the opportunity to significantly improve the document. Below, we provide a point-by-point response.

**Comment**: The manuscript addresses an emerging and timely topic—smart restaurant research—through a bibliometric lens, offering practical implications for future development. However, the theoretical framework remains underdeveloped. A clearer integration of smart restaurant technologies with theories from digital economics, user experience design, and service management would significantly strengthen the academic contribution.

**Response**: Two paragraphs were added to the introduction addressing the contribution of theories to the academic field.

**Comment**: The visualization of the co-occurrence network and thematic clusters is a strength of the paper. However, the manuscript lacks validation of the clustering quality—no modularity value, silhouette coefficient, or statistical justification is reported. Such metrics would enhance the robustness of the thematic structure.

**Response**: A limitation was added regarding the lack of quantitative validation using metrics such as modularity or silhouette coefficient.

**Comment**: The review covers a wide range of subfields—AI, IoT, robotics, and service automation—but reads as a collection of isolated summaries. Organizing the literature around thematic pillars such as “Intelligent Services,” “Customer Interaction,” and “Operational Efficiency” would improve narrative flow and analytical depth. The manuscript highlights that most publications originate from countries such as China, India, and the United States. However, cross-national differences in regulatory environments, cultural acceptance, or technological infrastructure are not elaborated upon. Case-based comparative insights would enrich the analysis and global relevance.

**Response**: The introduction was organized coherently. The analysis of benchmarks was improved by delving into how regulatory policies, technological infrastructure, and cultural acceptance influence the adoption of technologies in smart restaurants. An additional paragraph was created to complement the practical implications.

# Reviewer report: Reviewer response for version 2

**DOI:** 10.5256/f1000research.178017.r372421

**Published:** 2025-04-11

**Authors:** Li Ding

**Affiliations:** 1 Institut Lyfe, Ecully, France

The research topic is interesting and valuable.

 The introduction section is well written with the cutting-edge applications of technologies in the restaurant industry. It’s better to provide the formal definition of “smart restaurant” in this paper.

 In the methods section, when setting up a search strategy, why VR and AR technologies were considered smart restaurants? Again, here it needs to define the scope of the smart restaurant. In addition, the time window should be mentioned. The languages of the articles should be mentioned.

 In the results section, all results are presented well with figures and explanations. One question from the disciplinary and thematic analysis is, as the top cited journal is management-focused, however, there are no management-related themes identified from the annual main thematic development results. It’s interesting to know. 

 The conclusion and discussion sections are well written with sufficient information and implications. For the writing style, it’s better to combine some short paragraphs based on the meaning and structure. Currently, there are too many short paragraphs (less than four lines). 

 Overall, the quality of communication is high. I’m very happy to review this high-quality manuscript. Congratulations!

Is the work clearly and accurately presented and does it cite the current literature?

Yes

If applicable, is the statistical analysis and its interpretation appropriate?

Yes

Are all the source data underlying the results available to ensure full reproducibility?

Yes

Is the study design appropriate and is the work technically sound?

Yes

Are the conclusions drawn adequately supported by the results?

Yes

Are sufficient details of methods and analysis provided to allow replication by others?

Yes

Reviewer Expertise:

Hospitality and Restaurant Management; AI business adoption in hospitality and restaurant industry

I confirm that I have read this submission and believe that I have an appropriate level of expertise to confirm that it is of an acceptable scientific standard.

# Reviewer report: Reviewer response for version 2

**DOI:** 10.5256/f1000research.178017.r367040

**Published:** 2025-03-17

**Authors:** Rainer Alt, Gräser Gräser

**Affiliations:** 1 Leipzig University, Leipzig, Germany; 2 Information Systems Institute, Leipzig University, Leipzig, Saxony, Germany

Dear authors,

 Thank you for the opportunity to review the revised version of the manuscript "Trends in Smart Restaurant Research: Bibliometric Review and Research Agenda". Although I recognize that you have addressed some of my prior concerns (correcting formal issues and improving the discussion), I still have concerns regarding the study's design and execution, particularly concerning the search strategy and the interpretation of results.

 First, the search strategy is not convincing and in my opinion undermines the entire study. This results in an incomplete and biased data set, making the conclusions unreliable. It pertains particularly regarding the incompletely formulated search terms as well as the inclusion and exclusion criteria. You justify the incomplete search term with: “The divergence in each equation was justified”. However, it is clearly not justified, since certain areas appear cherrypicked (why include AI and not ML if ML is a major topic in the findings/discussion, why are some words included in full lengths with abbreviations and others not (why iot and not vr) and why are search terms for title and keywords different ((iot AND restaurant*) vs (“IoT AND restaurant*”)?).

 Second, and partly related with the incomplete inclusion and exclusion criteria, is the identification of the “Chinese Restaurant Process” (CRP) as a relevant topic in smart restaurant research. I still wonder why CRP should be relateda to smart restaurant research explicitly since it is a concept from probability theory (see for example https://www.cs.princeton.edu/courses/archive/fall07/cos597C/scribe/20070921.pdf). Problems like CRP accidentally contain the same word “restaurant” and the theory has been illustrated with examples from the restaurant industry, but CRP is not exclusive to smart restaurants. Following your argumentation to keep CRP (reinforcement learning algorithm for decision-making), you would also need to include papers with other algorithms that could be used for decision-making. Another example of papers which is questionable is “Discrete event simulation in prolog”. Of course, you can code applications for restaurants in Prolog but you can also code them in Python. Then, you should include books about Python in your SLR as well. I hope this makes the issues with the methodological approach visible.

 Referring to your response: “Could you please specify the point where this mention is made, in order to make the recommended adjustments”: The SLR comment refers to the section “Analysis of the thematic development of smart restaurants”

 Due to my methodological concerns I regret that I cannot recommend acceptance at this stage. However, I wish you all the best in further developing your research and hope that my feedback is helpful in this endeavor.

Is the work clearly and accurately presented and does it cite the current literature?

Partly

If applicable, is the statistical analysis and its interpretation appropriate?

Not applicable

Are all the source data underlying the results available to ensure full reproducibility?

Partly

Is the study design appropriate and is the work technically sound?

Partly

Are the conclusions drawn adequately supported by the results?

Partly

Are sufficient details of methods and analysis provided to allow replication by others?

No

Reviewer Expertise:

Business information systems, Electronic Business, Digital Platforms

We confirm that we have read this submission and believe that we have an appropriate level of expertise to state that we do not consider it to be of an acceptable scientific standard, for reasons outlined above.

# Reviewer report: Reviewer response for version 1

**DOI:** 10.5256/f1000research.173609.r355093

**Published:** 2025-02-03

**Authors:** Rainer Alt, Gräser Gräser

**Affiliations:** 1 Leipzig University, Leipzig, Germany; 2 Information Systems Institute, Leipzig University, Leipzig, Saxony, Germany

Thank you for the opportunity to review your paper on restaurant technologies, which I consider as highly relevant, given the increasing digitalization of the hospitality sector and the potential benefits of technological advancements for operational efficiency and customer experience in this domain. I agree that a well-conducted systematic literature review could provide valuable insights into current research trends, gaps, and future directions. However, despite the significance of this research area, the present paper suffers from substantial methodological shortcomings, a discussion section that fails to engage critically with the findings, and formal writing issues. Due to these concerns, the paper should not be recommended for indexed in its current form.

**1. Methodology**

 First, the literature review yields only a small set of publications that is not sufficient for claiming a systematic review in the smart restaurant sector. Second, the validity of the literature review is limited since the exclusion criteria are not clearly defined, leading to ambiguity in how studies were selected. For instance, the phrase "Only systematic literature reviews are subject to the second phase of exclusion, which involves eliminating all documents for which the full text is not available" on page 5 raises concerns about whether studies without full-text access were still included in the first phase. Third, the selection of databases (Web of Science and Scopus) raises questions about completeness. While these databases are highly regarded, they may not cover all relevant publications in the field of smart restaurant technologies. Fourth, the search strings differ between Scopus and WoS, which could lead to inconsistent results. The inclusion of separate search strings in uppercase and lowercase is unnecessary, as search engines are generally case-insensitive. In addition, the selection of technologies appears arbitrary, including some while excluding others without justification (no robots, machine learning etc.) and the use of abbreviations in the search string appears inconsistent (why not "AI" as an abbreviation). Fifth, elements such as assessment for reporting bias and study of risk bias assessment add little value to the argumentation, as they are borrowed from medical research and do not fit the context of this study. On the other hand, important methodological details such as the exact time frame of the review are missing, significantly undermining the study’s credibility.

**2. Contribution**

 A well-structured discussion section should critically engage with the findings. However, in this paper, the discussion is largely a repetition of the results without meaningful interpretation or critique. The first part of the discussion, in particular, reads more like a continuation of the results section rather than an analysis of what these findings mean. Additionally, certain elements in the discussion raise questions about the selection and categorization of studies. The paper refers to "systematic literature reviews" (SLR) as a "concept of smart restaurants," but SLRs are a methodological approach, not a conceptual framework. The inclusion of the "Chinese Restaurant Process" (CRP), which is a mathematical theorem, is questionable. One of the identified papers concerning CRP, titled "A Sample Aggregation Approach to Experiences Replay of Dyna-Q Learning," does not appear to be directly related to smart restaurant research. This suggests problems in properly excluding irrelevant studies. The lack of critical engagement in the discussion significantly weakens the paper’s contribution to the field. Rather than simply reiterating the findings, the discussion should aim at obtaining deeper insights and possibly also a more elaborate research agenda (see page 19).

**3. Formal issues**

 From the formal side, the paper requires thorough proofreading and editing. The excessive number of paragraphs disrupts readability and cohesion, making certain sections appear fragmented. For instance, the sentences "Technology adoption in the restaurant sector remains low due to a variety of interrelated factors. first, resistance to change by restaurant owners and managers can be a significant barrier, many of which may be rooted in traditional practices. and may be reluctant to invest in technology due to concerns about cost, implementation complexity, and fear of operational disruption. and a lack of knowledge and understanding of how to effectively integrate technology into restaurant operations can lead to uncertainty and avoidance. adoption." on page 3 contains multiple abrupt transitions. Additionally, the inconsistent use of hyphenation, such as "ap-propriate" on page 4 indicates a lack of standardization in formatting. The term "Restoration" is used on page 12 instead of "restaurants," which is misleading and likely a typographical error. On page 5, the phrase "[...] current restaurant bibliometrics. intelligent" is incomplete. These formal errors create the impression of an unfinished manuscript and hinder comprehension, reducing the overall professionalism of the paper.

 I trust that the authors find this feedback helpful and wish them all the best in further developing their work.

Is the work clearly and accurately presented and does it cite the current literature?

Partly

If applicable, is the statistical analysis and its interpretation appropriate?

Not applicable

Are all the source data underlying the results available to ensure full reproducibility?

Partly

Is the study design appropriate and is the work technically sound?

Partly

Are the conclusions drawn adequately supported by the results?

Partly

Are sufficient details of methods and analysis provided to allow replication by others?

No

Reviewer Expertise:

Business information systems, Electronic Business, Digital Platforms

We confirm that we have read this submission and believe that we have an appropriate level of expertise to state that we do not consider it to be of an acceptable scientific standard, for reasons outlined above.

# Response

**Published:** 2025-02-05

**Authors:** JHOANY ALEJANDRO VALENCIA ARIAS

Kind regards,

 Dear reviewer, we have submitted the latest version of the manuscript, addressing all the suggestions you have made. We will respond to your suggestions one by one. The journal will send you the updated version.

**comment**: Thank you for the opportunity to review your paper on restaurant technologies, which I consider as highly relevant, given the increasing digitalization of the hospitality sector and the potential benefits of technological advancements for operational efficiency and customer experience in this domain. I agree that a well-conducted systematic literature review could provide valuable insights into current research trends, gaps, and future directions. However, despite the significance of this research area, the present paper suffers from substantial methodological shortcomings, a discussion section that fails to engage critically with the findings, and formal writing issues. Due to these concerns, the paper should not be recommended for indexed in its current form.

**Response**: Several sections of the discussion were improved by incorporating a more critical analysis

**comment: **1. Methodology

 First, the literature review yields only a small set of publications that is not sufficient for claiming a systematic review in the smart restaurant sector.

**Response**: A paragraph was added to the limitations justifying the number of studies included in the research

**comment**: 1. Methodology

 Second, the validity of the literature review is limited since the exclusion criteria are not clearly defined, leading to ambiguity in how studies were selected. For instance, the phrase ""Only systematic literature reviews are subject to the second phase of exclusion, which involves eliminating all documents for which the full text is not available"" on page 5 raises concerns about whether studies without full-text access were still included in the first phase.

**Response**: The inclusion and exclusion process was clearly explained in the methodology

**comment**: 1. Methodology

 Third, the selection of databases (Web of Science and Scopus) raises questions about completeness. While these databases are highly regarded, they may not cover all relevant publications in the field of smart restaurant technologies.

**Response**: The choice of the databases was justified

**comment**: 1. Methodology

  Fourth, the search strings differ between Scopus and WoS, which could lead to inconsistent results. The inclusion of separate search strings in uppercase and lowercase is unnecessary, as search engines are generally case-insensitive. In addition, the selection of technologies appears arbitrary, including some while excluding others without justification (no robots, machine learning etc.) and the use of abbreviations in the search string appears inconsistent (why not ""AI"" as an abbreviation).

**Response**: The divergence in each equation was justified

**comment**: 1. Methodology

 Fifth, elements such as assessment for reporting bias and study of risk bias assessment add little value to the argumentation, as they are borrowed from medical research and do not fit the context of this study. On the other hand, important methodological details such as the exact time frame of the review are missing, significantly undermining the study’s credibility.

**Response**: The inclusion of the effect measures sections was justified. The reporting bias section was removed

**comment**: 2. Contribution

 A well-structured discussion section should critically engage with the findings. However, in this paper, the discussion is largely a repetition of the results without meaningful interpretation or critique. The first part of the discussion, in particular, reads more like a continuation of the results section rather than an analysis of what these findings mean.

**Response**: The discussion section of the results was addressed more critically

**comment**: 2. Contribution

  Additionally, certain elements in the discussion raise questions about the selection and categorization of studies. The paper refers to ""systematic literature reviews"" (SLR) as a ""concept of smart restaurants,"" but SLRs are a methodological approach, not a conceptual framework.

**Response**: Could you please specify the point where this mention is made, in order to make the recommended adjustments

**comment**: 2. Contribution

 The inclusion of the ""Chinese Restaurant Process"" (CRP), which is a mathematical theorem, is questionable. One of the identified papers concerning CRP, titled ""A Sample Aggregation Approach to Experiences Replay of Dyna-Q Learning,"" does not appear to be directly related to smart restaurant research. This suggests problems in properly excluding irrelevant studies.

**Response**: The inclusion of the citation is justified. This article is relevant in the field of reinforcement learning algorithms and is known for its introduction to the Dyna-Q algorithm, which combines planning and experience-based learning, which can be related to intelligent decision-making systems.

**comment**: 2. Contribution

 The lack of critical engagement in the discussion significantly weakens the paper’s contribution to the field. Rather than simply reiterating the findings, the discussion should aim at obtaining deeper insights and possibly also a more elaborate research agenda (see page 19).

**Response**: This comment was addressed by improving the discussion

**comment**: 3. Formal issues

 From the formal side, the paper requires thorough proofreading and editing. The excessive number of paragraphs disrupts readability and cohesion, making certain sections appear fragmented. For instance, the sentences ""Technology adoption in the restaurant sector remains low due to a variety of interrelated factors.

**Response**: The paragraph was improved by correcting the sentence

**comment**: 3. Formal issues

 first, resistance to change by restaurant owners and managers can be a significant barrier, many of which may be rooted in traditional practices. and may be reluctant to invest in technology due to concerns about cost, implementation complexity, and fear of operational disruption. and a lack of knowledge and understanding of how to effectively integrate technology into restaurant operations can lead to uncertainty and avoidance. adoption."" on page 3 contains multiple abrupt transitions.

**Response**: The wording of the introduction was improved, specifically page 3

**comment**: 3. Formal issues

 Additionally, the inconsistent use of hyphenation, such as ""ap-propriate"" on page 4 indicates a lack of standardization in formatting. The term ""Restoration"" is used on page 12 instead of ""restaurants,"" which is misleading and likely a typographical error. On page 5, the phrase ""[...] current restaurant bibliometrics. intelligent"" is incomplete.

**Response**: The word separation was corrected. The incorrect word and sentence were also corrected.

 We hope that these corrections will best respond to your kind suggestions,

 --

 The authors
